# Increased microclimatic variation in artificial nests does not create ecological traps for a secondary cavity breeder, the European roller

**DOI:** 10.1002/ece3.6871

**Published:** 2020-11-18

**Authors:** Timothée Schwartz, Arnaud Genouville, Aurélien Besnard

**Affiliations:** ^1^ A Rocha France, Domaine des Courmettes Tourrettes‐sur‐Loup France; ^2^ Institut de recherche de la Tour du Valat Arles France; ^3^ CEFE, Univ Montpellier, CNRS, EPHE‐PSL University, IRD, Univ Paul Valéry Montpellier 3 Montpellier France; ^4^ UniLasalle Mont‐Saint‐Aignan France

**Keywords:** buffering capacity, *Coracias garrulus*, humidity, natural cavities, nest box, preference, temperature

## Abstract

Artificial devices are increasingly used in conservation measures to mitigate the disappearance of natural habitats. However, few studies have demonstrated their benefits for the target species, and they may pose a risk of creating ecological traps. This occurs when lower individual fitness is found in artificial habitats that are more attractive than their natural equivalents. In this study, we tested the ecological trap hypothesis on a dense population of European rollers *Coracias garrulus* breeding in both natural cavities and nest boxes. Our initial prediction was that the more stressful microclimatic conditions of nest boxes would lead to reduced fitness of European rollers, thus creating an ecological trap. The results showed that nest boxes were preferred over natural cavities. Despite significantly more extreme microclimatic conditions in nest boxes, we found similar breeding parameters between artificial and natural nest types. Our results also suggest that European rollers selected the nest boxes which best buffered the temperature, thus avoiding potential ecological traps. Overall our results led to the conclusion that nest boxes do not create ecological traps for European rollers in this study area. However, other species may be more sensitive to microclimatic variations or less able to avoid the least favorable nest boxes. These findings could help to inform the placement of nest boxes in order to reduce extreme temperatures and variation in humidity rates. Future studies could compare nest types for other fitness parameters, such as juvenile body condition or survival. We also recommend the ecological trap hypothesis as a useful framework to evaluate the outcomes of artificial devices used for conservation.

## Graphical abstract

Artificial devices are increasingly used in conservation, but few studies have demonstrated their benefits for the target species, while they may pose a risk of creating ecological traps. Here, by comparing occupation, breeding parameters, and microclimate with natural cavities, we demonstrate that nest boxes are not ecological traps for European rollers *Coracias garrulus* in South of France, despite significantly more extreme microclimatic conditions. Our findings also suggest that rollers selected the nest boxes which best buffered the temperature, thus avoiding potential ecological traps.

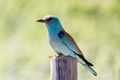

## 1 INTRODUCTION

The rapid changes affecting biodiversity in recent decades have led to an expansion of habitat restoration and biodiversity offsetting programs (Dobson, 1997; Maron et al., 2015). These initiatives have resulted in the development of numerous methods to restore or recreate habitats that have been damaged or destroyed by human activities such as urbanization or intensive agriculture. Of these methods, the use of artificial devices for conservation is increasingly implemented as a response to habitat degradation. For example, shelters for reptiles are positioned to replace disappearing stone walls and hedges (Grillet et al., 2010), bat boxes to compensate for felled cavity trees or restored buildings (Flaquer et al., 2006), and nest boxes to provide additional breeding places for birds and mammals (Goldingay & Stevens, 2009). Yet despite their extensive use in conservation and offsetting programs, robust evaluations of the benefit of artificial devices in terms of the population viability of the target species are rare (Wesołowski, 2011, but see Bourgeois et al., 2015; Bragin et al., 2017; Libois et al., 2012; Sutherland et al.., 2014). The success of artificial devices is generally evaluated using single indicators, for example, colonization by the target species, (Aleman & Laurens, 2013; Avilés & Sanchez, 2000; Chapman & Blockley, 2009) or, in the best cases, their impact on some selected demographic parameters, such as breeding success or survival (Bourgeois et al., 2015; Libois et al., 2012). However, measuring occupation alone does not necessarily demonstrate any benefit for the population. A fundamental issue is that while an artificial site may be attractive to the target species, it may have a deleterious impact on reproduction: for instance, by increasing predation risk (Robertson & Hutto, 2006). Furthermore, positive outcomes for the population are difficult to demonstrate unless demographic parameters (for instance, fecundity) in artificial devices are compared with those in natural breeding sites. Several studies comparing artificial devices alone in different contexts have shown that the fitness of the target species can decrease in some situations, such as inappropriate nest size (Demeyrier et al., 2016) or nest placement (Rodríguez‐Ruiz et al., 2011), hence creating an ecological trap (Klein et al., 2007).

**Figure 1:**
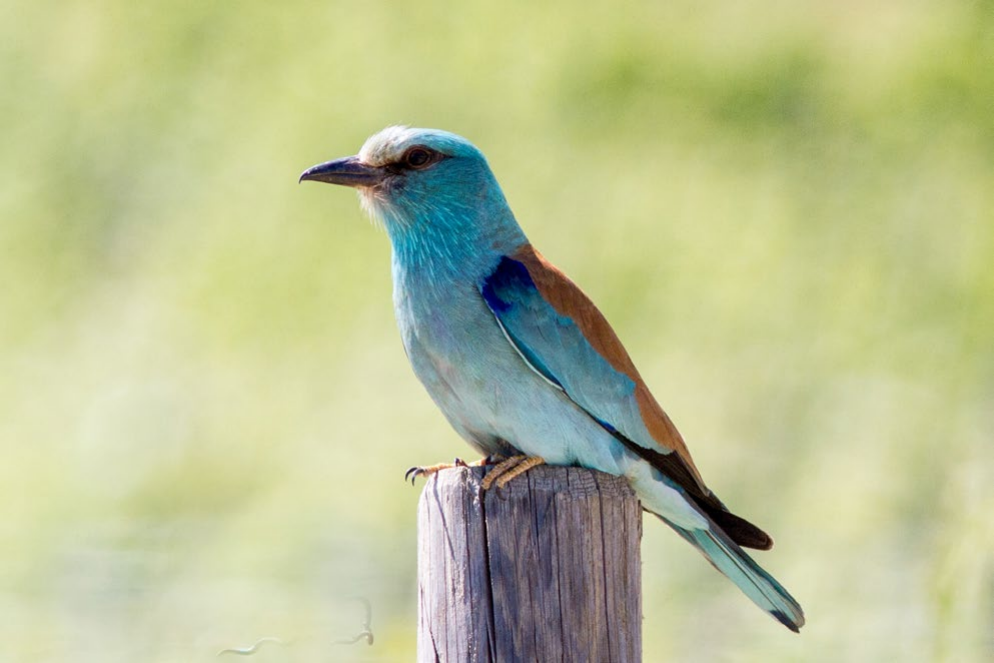
European roller *Coracias garrulus* in Vallée des Baux, France (Author: Peter Harris)

An ecological trap is a modified habitat that is preferred by a target species over an unmodified habitat, but which reduces the fitness of individuals (Robertson & Hutto, 2006). Ecological traps generally occur when perceived habitat quality of the modified habitat does not match its actual quality (Schlaepfer et al., 2002). In the context of artificial devices, while some evaluations only explore either its attractiveness or its impact on the fitness of the target species, testing the ecological trap hypothesis requires to examine both (Robertson & Hutto, 2006). It also relies on comparing these parameters between the artificial device and the unmodified habitat that it aims to mimic. This latter aspect is, however, often lacking in evaluations of artificial conservation tools (but see Bourgeois et al., 2015). The test of the ecological trap hypothesis is thus a two‐steps process (Figure 1). First, studying a species’ preference for artificial or natural habitats (or components of the species’ habitat) is essential as avoidance of artificial devices would lead to the failure of a conservation strategy. Avoidance means that the target species is less attracted by the artificial habitat compared with its natural habitat and is generally the result of a lack of knowledge of the drivers of the target species’ habitat selection needed for relevant device conception or placement (Battin, 2004; Robertson & Hutto, 2006). The second step consists in measuring fitness parameters or proxies at the population level (for example, demographic parameters), which is often more challenging than for preference. These parameters must be indeed compared with known parameters for healthy populations of the same species living in an unmodified habitat in a similar context, however, such habitats are often difficult to find or access (Lambrechts et al., 2010).

Artificial shelters for cavity‐using species, such as nest boxes for birds or bats, are good models for testing the ecological trap hypothesis. They are probably the most widely used artificial devices for species conservation (Goldingay & Stevens, 2009). Their popularity among conservationists is mostly due to their low cost and the ease with which they can be deployed and surveyed (Hayward et al., 1992; Lambrechts et al., 2010). They are used in conservation programs to respond to the growing disappearance of natural cavities in natural and semi‐natural environments because of intensive forest management and landscape simplification (Lindenmayer et al., 2009). However, numerous studies have reported potential differences between natural cavities and artificial nest boxes (reviewed in Lambrechts et al., 2010; Møller, 1989; Wesołowski, 2011) such as predation risk (Fargallo et al., 2001), parasite load (Amat‐Valero et al., 2014), or microclimate (Maziarz et al., 2017). These potential differences are critical because numerous factors affect preference and fitness of species using cavities (see reviews in Goldingay & Stevens, 2009; Lambrechts et al., 2010), predation risk (Fontaine & Martin, 2006; Mönkkönen et al., 2009; Wesołowski, 2002; but see Lima, 2009; Martin, 1993; Pöysä et al., 2001 for absence of effect on preference) and microclimate (Rhodes et al., 2009; Wachob, 1996) being among the most important for cavity selection. Ambient temperature and humidity within breeding cavities are key drivers of incubation and chick development (Mersten‐Katz et al., 2012; Mueller et al., 2019; Wesołowski, 2011), which can lead parents to select the most climatically favorable breeding environments (Rhodes et al., 2009). In artificial devices, the microclimate is not always as stable as in natural cavities; previous studies have shown that extreme temperatures in artificial boxes could impact the fitness of its occupants, for example, in bats (Flaquer et al., 2006) or birds (Catry et al., 2015).

**Figure 2:**
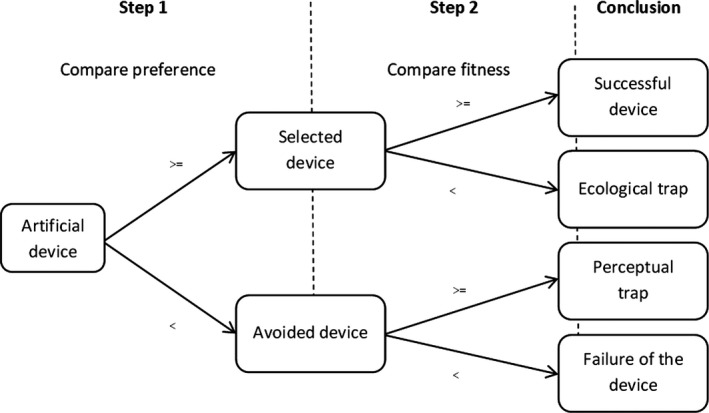
Conceptual framework for evaluating artificial conservation devices based on the ecological trap hypothesis: “>=” means values for artificial device are higher or equal compared to values for unmodified habitat; “<” means values for artificial device are lower compared to unmodified habitat

In this study, we took advantage of a dense population of European rollers *Coracias garrulus* (Figure 2), breeding both in natural cavities and nest boxes, to formally test the ecological trap hypothesis for an artificial conservation device. To do this, we compared occupation rates and breeding success of nest boxes and natural cavities and investigated the impact of microclimate on breeding success and occupation probability.

The European roller is an obligate secondary cavity breeder (Cramp, 1985), it does not create or modify its breeding site but uses already available sites on arrival after migration (Cramp, 1985). Thus, European rollers respond relatively quickly to nest‐box deployment (Aleman & Laurens, 2013) sometimes by abandoning nearby natural cavities in favor to nest boxes (Valera et al., 2019) and as such are very good models for studying nesting site preference. Because of this behavior, we expected higher occupancy rates in nest boxes compared with natural cavities. To our knowledge, no previous study compared occupancy between natural and artificial nests for European rollers. We expected artificial nests to have less microclimatic buffering capacity than natural nests because of structural differences (Amat‐Valero et al., 2014; Catry et al., 2015; Maziarz et al., 2017), and that these more variable conditions would lower breeding performance, following Catry et al. (2015) who found that European roller chicks’ mass gain decreased with extreme heat events in nest boxes but not in adobe wall cavities. Based on these two predictions, we expected nest boxes to act as an ecological trap for the European roller.

## 2 MATERIAL AND METHODS

### 2.1 The European roller

The European roller is a trans‐Saharan migratory bird that winters in southern Africa (Finch et al., 2015) and breeds between May and July in a range that extends from the Mediterranean basin through Eastern Europe to central Asia (Cramp, 1985). Breeding sites are principally woodpecker cavities, but also include bee‐eater burrows in sand cliffs and cavities in buildings such as farmhouses or bridges (Cramp, 1985). European rollers do not build their own nest and occupy readily available cavities (Cramp, 1985). As such they do not modify the structure of the nesting site, and hence do not actively modify the internal microclimate of cavities, other than by their own presence inside the nest. Despite its recent re‐ranking to “least concern” on the global IUCN Red List (IUCN, 2016), the European roller remains a threatened species in most European countries (Tokody et al., 2017). European rollers have benefited from numerous conservation programs over the past two decades (Tokody et al., 2017), including the deployment of thousands of nest boxes all over Europe (Finch et al., 2019).

### 2.2 Study area

The study took place in the valley of Les Baux‐de‐Provence (43°41′N, 4°46′E; WGS 84), located near the city of Arles, in the Bouches du Rhônes department of France. The region is classified as a meso‐mediterranean bioclimatic stage, with hot and dry summers (average maximum temperature of 30°C in July). The valley extends over 2,000 ha of cereal fields and grasslands. A dense drainage channels’ network extending throughout the valley is bordered by several kilometers of hedges and riparian forest (Chambre d’Agriculture des Bouches du Rhône, 2008), where black poplars *Populus nigra* and white poplars *Populus alba* offer an exceptional density of natural cavities, created mostly by European green woodpeckers *Picus viridis* (Butler, 2001). This situation contrasts with other areas in southern France, where availability of nesting sites has been recently confirmed as a major limiting factor in European roller breeding pairs’ density (Finch et al., 2019). Common starling *Sturnus vulgaris* is the main competitor of European rollers for cavities in the study area, but breeds averagely earlier and is actively chased from the nesting sites by European roller pairs at their arrival (A Rocha France, unpublished data).

### 2.3 Nest monitoring

We monitored a network of 50 nest boxes, which were deployed in the study area for the study and conservation of European rollers (Figure S1). The nest boxes were built using plywood (15 mm thick) and had a volume of ~21,870 cm^3^ (Height of the front wall = 25 cm; Height of the back wall = 35cm; Length of the side walls = Width of the front and back walls = 27 cm) and 57–60 mm entrance hole. They were installed on trees at different heights and orientations. We maintained and cleaned (removing of old nest material) all nest boxes on a yearly basis. Nest‐box occupation has been checked at least three times every year from mid‐May to the end of June between 2016 and 2019, using a Voltcraft BS‐250XIPSD endoscopic camera mounted on a telescopic pole. In breeding situations, at least three additional inspections were organized at c.a. one‐week intervals to survey the number of laid eggs, hatched eggs, and fledglings per nest. All fledglings were ringed at between 15 and 25 days old. Fledging success was determined on the basis of the number of fledglings ringed, but was corrected retrospectively if a chick died after ringing and was found during the nest‐box cleaning session. The date of the first egg laying was back‐calculated from nest monitoring observations (using two‐days interval between the laying of each egg and 21 days between first egg‐laying date and hatching (Personnal field obs. and Guillaumot, 2016)). Nest abandonments and predation events (defined as the observation of damaged or disappeared eggs, or the presence of killed individuals in the nest) were also systematically recorded.

Between 2016 and 2019, a sample of accessible natural cavities located on the same study area was checked for occupation by European rollers. Monitoring of the natural cavities was similar to that described for nest boxes only in 2017. In other years, monitoring intensity was generally less frequent, and hence breeding parameters were only calculated for occupied nests visited at least three times between first egg‐laying and first chick‐fledging dates. For both nest types, European roller breeding monitoring occurred between the 20 May and the 17 August.

In 2017, we explored the study area intensively and located 190 natural cavities suitable for European roller reproduction on 154 different trees (Figure S1). Suitability of cavities for European roller reproduction was determined based on minimal hole diameter of occupied cavities at our study area (47 mm) (A Rocha France, unpublished data) and location in the tree (no branches in front of the cavity hole enabling access for predators), all other tree and cavity characteristics being not selected by European rollers in the study area (see previous studies Butler, 2001; Dasse, 2016; Eltabet, 2013). For all occupied (*n* = 23) and a sample of randomly selected unoccupied cavities (*n* = 77) accessible by foot or with a 8 m ladder, we measured height above ground, circumference of the tree at breast height (1.30 m) and at cavity height, orientation, entrance height, entrance width, depth, and length (Table 1).

**Table 1:** Name and description of the different microclimate and nest variables measured in natural cavities and nest boxes in the Vallée des Baux (France).

	Name		
	Daily variable	Mean variable over 15 consecutive days	Description
Microclimate variables	MaxTint	MAXTint	Maximal temperature in the nest (°C)
	MinTint	MINTint	Minimal temperature in the nest (°C)
	MoyTint	MOYTint	Mean temperature in the nest (°C)
	DiffTint	DIFFTint	Difference between maximal and minimal temperature in the nest (°C)
	Dtmoyabs	DTMOYabs	Mean over a day of the absolute delta between interior and exterior temperature (°C)
	Dtmaxabs	DTMAXabs	Absolute value of the daily maximum delta between interior and exterior temperature (°C)
	Maxhum	MAXHum	Maximum humidity level in the nest (%RH)
	Minhum	MINHum	Minimum humidity level in the nest (%RH)
	Moyhum	MOYHum	Mean humidity level in the nest (%RH)
	DiffHum	DIFFHum	Difference between maximal and minimal humidity level in the nest (%RH)

	Name	Name of the nest box or cavity
Nest variables	Type	Nest type: either nest box or cavity
	occupation	Occupation of the nest by rollers during the breeding season: 1 for roller nests, 0 otherwise
	OccR	Real occupation of the nest during monitoring (roller or starling): 1 for occupied nests sensor measurements, 0 for empty nests during sensor measurements
	Developmental stage	Number of days between the day of measurement and the first egg‐laying date (0 for nests not occupied by rollers)
	First egg‐laying date	Day of the laying of the first egg (for roller nests only) (Julian calendar date)
	CH	Cavity height (m)
	CBH	Circumference of the tree at breast height (1.30m) (cm) (natural cavities only)
	CCH	Circumference of the tree at cavity height (cm) (natural cavities only)
	Orientation	Entrance orientation of the nest (degrees)
	Ori	Simplified orientation of the nest (either *N*, E, S or W with *N*: 316°−45°; E: 46°−135°; S: 136°−225°; W: 226°−315°)
	EH	Entrance height (cavity) (mm) (natural cavities only)
	EW	Entrance width (cavity) (mm) (natural cavities only)
	CL	Cavity length (cm) (vertical): minimum distance from the bottom of entrance hole to the bottom of the chamber (natural cavities only)
	CD	Cavity depth (cm) (horizontal): minimum distance from the entrance hole to the back wall of the cavity (natural cavities only)
Other variables	Date	Day of the measurement (Julian calendar date)

Note

“%RH”: percentage of relative humidity.

### 2.4 Microclimate monitoring

In 2017, we monitored the microclimate in the nest boxes (*n* = 17) and natural cavities (*n* = 23) occupied by European rollers, as well as in a sample of randomly selected nest boxes (*n* = 16) and natural cavities (*n* = 18) empty or occupied by common starlings (Table S1). Many nests occupied by European rollers are initially occupied by starlings, and rollers either wait until the fledging of starling chicks or chase them out before occupying the nesting site (Cramp, 1985). Therefore, cavities occupied by starlings are potential breeding sites for rollers and were included in the sample. Orientation and height of monitored nest boxes did not differ from available nest boxes (Orientation: Chi^2^ = 0.18, *df* = 3, *p = *.98; height: *F*_1,71_ = 0.19, *p = *.66). Monitored natural cavities had the same orientation but were in average 1.05 m higher than the sample of measured available cavities (Orientation: Chi^2^ = 1.14, *df* = 3, *p = *.77; height: *F*_1,133_ = 4.98, *p = *.03), respectively. Interior temperature (°C) and humidity (percentage of relative humidity: %RH), and exterior temperature (°C) were, respectively, measured with iButtons DS1923 and DS1921G‐F5 (Maxim Integrated Products, Inc.). Measurements were taken every hour for 15 consecutive days during the period between 6 June 2017 and 20 August 2017, which covers most of the incubation and chick‐rearing period in our study area (23 May–10 August, based on 18 years of European roller breeding monitoring on the study area, (A Rocha France, unpublished results)). For occupied nests, sensors were deployed only after the clutch was complete, in order to prevent nest abandonment. In the nest boxes, interior sensors were screwed directly to the rear wall, with the captor facing inside the box, and in the cavities, sensors were placed inside the hole at least 20 cm away from the entrance and held by a flexible aluminium angle bracket screwed directly into the bark of the tree. The aluminium rods were restricted to the sides of the cavity entrance in order to reduce possible disturbance of animal movements in and out of the cavity. Exterior sensors were placed in the shade, either under the base of the nest boxes, facing the ground, or on the bark of the tree at cavity height, with the captor facing north. As occupied nests were discovered at different developmental stages (from egg incubation to chick rearing), sensor deployment periods in occupied nests covered different stages among and within the nests (from incubation to postfledging stages). The occupation of each nest by European rollers or common starling (the only other species using the monitored nest boxes and cavities during our study) during the measurement period was recorded.

The IButton data were extracted with OneWireViewer software (1‐Wire drivers X86, version 4.03). The humidity data were corrected following the sensor manufacturer's recommendations (Maxim Integrated Products Inc, 2011: p. 53). For each nest and each day, we calculated: (a) the maximum, minimum and mean interior temperature and humidity rate, (b) the daily amplitude of the interior temperature and humidity (i.e., the difference between the minimum and maximum daily temperature/humidity rate inside the nest), (c) the daily mean and maximum absolute delta between the interior and exterior temperature (i.e., the mean and the maximum over 24h of the absolute values of hourly differences between exterior ambient temperature and interior nest temperature). Additionally, we calculated (d) the mean value over 15 consecutive days of each of the above parameters (Table 1).

## 3 STATISTICAL ANALYSES

### 3.1 Nest type preference

We measured the preference for nest types (natural vs. artificial nests) in 2017 using the estimated occupancy rate (Johnson, 1980) and the first egg‐laying date (Julian calendar date) as a proxy for settlement date of the European rollers at nesting site (Robertson & Hutto, 2006). We considered only one cavity per tree in the sample (*n* = 154), as European rollers are territorial; to our knowledge, more than one European roller pair per tree has never been recorded. We compared occupancy rates between nest types using a Generalized Linear Model (GLM) with a binomial distribution and logit link function. However, as the sample included all nest boxes (*n* = 50) and occupied natural cavities (*n* = 23) but only an unknown proportion of the available natural cavities (*n* = 131) (inevitably lower than the actual amount), the true proportion of occupied natural cavities was lower than our estimate. We tested the effect of nest type on the 2017 first egg‐laying date using a Linear Model with nest type as the explanatory variable.

### 3.2 Breeding success

Ambient temperatures (Table S2) as well as breeding and foraging habitats (A Rocha France yearly habitat surveys, unpublished data) were very similar on the study area from 2016 to 2019 during the core of the European roller breeding season. Therefore, we included occupied nests that were intensively monitored (*n* = 69 and *n* = 64 for nest boxes and natural cavities, respectively) during the 2016–2019 breeding seasons for the breeding success analysis in order to increase the sample size. We tested the difference of predation frequency (over breeding attempts with known outcome) between nest types using a Chi^2^ test. Successful nests were defined as European roller nests with at least one fledgling. We tested the correlation between breeding success parameters and nest type using Generalized Linear Mixed Models (GLMMs) with nest type as fixed factor, breeding year and nest identity as random factors and three different breeding response variables (Poisson distribution and log link function for the number of eggs and number of fledglings per nest, binomial distribution and logit link function for the probability that a nest was successful). Finally, for successful nests only (*n* = 62 and *n* = 38 for nest boxes and natural cavities, respectively), we tested the correlation of the probability that an egg produced a fledging chick with nest type, using a GLMM with a binomial distribution and logit link function, with breeding year and nest identity as random factors. We checked the overdispersion in the residuals when using a Poisson distribution and none was detected.

### 3.3 Microclimate parameters

All the nest characteristic variables were scaled prior to analysis. We tested the correlation of nest characteristic variables with microclimate parameters. We used GLMMs with a Gaussian error structure, with the individual nest name (nest ID) and date (Julian calendar) as random factors. We compared cavity height and orientation between nest types, using a linear model with a Gaussian error structure and a Chi^2^ test, respectively. We tested the effect of nest height and orientation for both nest types on microclimate parameters. The models included the interaction of nest type (in order to control for nest characteristics disparities between nest types) and real occupation of the nest (i.e., nest occupation during sensor deployment) as additive factor (in order to control for the potentially induced modification of the microclimate (Maziarz, 2019; Veľký et al., 2010)) (see Section 4). All the other nest characteristics were available only for natural cavities and hence their correlation with microclimate was tested solely on the subset of natural cavities, with real occupation of the nest as additive factor in the models.

We compared the daily microclimate variables between nest types, using GLMMs with a Gaussian error structure, with nest name and date as random factors. We included the interaction of real occupation with nest type in order to control for the response to the presence of birds in the nest according to each nest type. We also included the Julian calendar date as additive covariate in order to control for possible increase or decrease of microclimate parameters over the course of the study, and lastly the number of days between the egg‐laying date of breeding European rollers and the day of measurement, in order to control for developmental stages of European rollers (in interaction with the occupation by European rollers in order to estimate the regression coefficients only for occupied nests). We performed a backward model selection procedure, starting from the full model and removing the factor with highest *p*‐value until all remaining factors had a *p*‐value <.05.

### 3.4 Effect of microclimate on breeding success and occupation probability

For the breeding parameters recorded during the 2017 breeding season alone, we tested the correlations of the mean microclimate variables over 15 consecutive days with (a) the number of eggs, (b) the number of fledglings, (c) the probability that a nest was successful, (d) the probability that an egg produced a fledging chick, and (e) the occupation probability by European rollers, using GLMs with a Poisson and log link (1,2) and binomial distribution and logit link (3,4,5), respectively. For each breeding parameter, we constructed models with a single microclimate variable, as most of them were correlated. For each microclimate variable, we controlled for possible disparities between nest types by including the interaction effect of nest type in the models.

All statistical analyses were performed with R environment version 3.4.3 (R Core Team, 2017) using packages nlme (Pinheiro et al., 2016), lme4 (Bates et al., 2015), and lmerTest (Kuznetsova et al., 2017).

## 4 RESULTS

### 4.1 Nest type preference

Occupancy rate of nest boxes by European rollers was significantly higher than that of natural cavities (0.34 95% CI [0.20;0.52] and 0.15 95% CI [0.10;0.21], respectively, *z* = 2.87, *df* = 1,203, *p < *.01). The first egg‐laying dates were similar for both nest types (146.6 95% CI [143.2;150.0] and 150.3 95% CI [145.3;155.2] for natural and artificial nests, respectively) (*F*_1,35_ = 2.14, *p = *.15).

### 4.2 Breeding success

The breeding parameter estimates of European rollers in the study area are summarized in Table 2. No significant difference was found between nest types for the probability that a nest was successful (*z* = 1.10, *df* = 1,3,114, *p = *.27) or for the probability that an egg produced one fledging chick in successful nests (*z* = −0.08, *df* = 1,3,86, *p = *.94). Nor was there a significant difference between nest types for average clutch size (*z* = 0.32, *df* = 1,3,101, *p = *.75) or mean number of fledglings (*z* = 1.38, *df* = 1,3,106, *p = *.17). 14 recorded breeding attempts failed during the 2016–2019 breeding seasons on the study area (7 in nest boxes and 7 in natural cavities) out of which 10 were predated, with no significant difference in frequencies between nest types (4 in nest boxes and 6 in natural cavities, Chi^2^ = 1.13, *df* = 1, *p = *.29).

**Table 2:** Estimates of breeding parameters for European rollers breeding in nest boxes or cavities between 2016 and 2019 in the Vallée des Baux (France) with corresponding 95% confidence interval and sample size (*n*)

	Nest boxes			Natural cavities			All nests		
	Estimate	95% CI	*n*	Estimate	95% CI	*n*	Estimate	95% CI	*n*
Probability that a nest was successful	0.91	[0.78–0.97]	69	0.85	[0.65–0.94]	46	0.87	[0.75–0.95]	115
Probability that an egg produced a fledging chick	0.85	[0.78–0.90]	62	0.85	[0.74–0.92]	25	0.85	[0.79–0.90]	87
Average clutch size	5.12	[4.60–5.70]	65	4.97	[4.30–5.75]	37	5.07	[4.65–5.52]	102
Average number of fledglings	3.83	[3.18–4.61]	69	3.28	[2.60–4.13]	38	3.64	[3.04–4.36]	107

### 4.3 Effect of nest characteristics on microclimate

Monitored natural cavities were significantly higher than monitored nest boxes (CH = 6.01 m 95% CI [5.32;6.70] for natural cavities, CH = 4.38 m 95% CI [3.35;5.41] for nest boxes) (*F*_1,61_ = 9.65, *p < *.01). The orientation of the nests monitored for microclimate differed between nest types (Chi^2^ = 11.34, *df* = 3, *p = *.01), with only one north‐facing nest box (3% versus 17% for natural cavities) and only two east‐facing nest boxes (7% vs. 29% for natural cavities) (Figure S2).

For both nest types, microclimate parameters were not correlated to nest height (Table S3a).

In natural cavities, western orientation had a slightly lower average and maximum temperature buffering capacity than southern orientation (Dtmoyabs: −1.43°C, *p = *.06; Dtmaxabs: −2.90°C, *p = *.05). Northern orientation had a slightly lower maximal temperature than southern and eastern (MaxTint: South: −1.99°C, *p = *.07; East: −1.89°C, *p = *.09) and a smaller interior temperature variation (DiffTint: South: −2.34°C, *p = *.03; East: −2.27°C, *p = *.04) (Figure S3a & b). Interior nest humidity was correlated to orientation, with north‐facing nests more humid than those oriented toward other directions (Maxhum: East: +9.75%RH, Minhum: +17.95 to +21.12%RH, Moyhum: +12.11 to +14.41%RH), with less variation in humidity (DiffHum: −9.57 to −14.57%RH) (Figure S3.C) (Table S3b).

In nest boxes, we compared only the western and southern orientations (because of small sample size for northern (*n* = 1) and eastern (*n* = 2) orientations) and found no differences between the two, except for maximum and average buffering capacity, which was significantly lower in the southern direction (Dtmoyabs: −0.40°C; Dtmaxabs: −0.98°C) (Table S3b).

For natural cavities only, maximum and average interior temperature decreased with the tree circumference at breast height (CBH) (MaxTint: *β_x_* = −0.85, *SE* = 0.42, *p = *.05; MoyTint: *β_x_* = −0.60, *SE* = 0.33, *p = *.08). Similarly, maximum interior temperature and interior temperature variation decreased with the tree circumference at cavity height (CCH) (MaxTint: *β_x_* = −0.80, *SE* = 0.37, *p = *.04; DiffTint: *β_x_* = −1.02, *SE* = 0.35, *p = *.01) (Figure S4.A & B). Cavities in thicker trunks or branches tended to be more humid, with minimum and average humidity rates slightly increasing with CCH (Minhum: *β_x_* = 4.92, *SE* = 2.61, *p = *.07; Moyhum: *β_x_* = 3.71, *SE* = 2.19, *p = *.099) (Figure S4.C) (Table S3a). Other nest characteristics were not correlated to any microclimate parameter (Table S3a).

### 4.4 Comparison of microclimate parameters between nest types and occupation status

Recorded interior and exterior temperatures for all nests ranged from 12.53°C to 43.45°C and 11.50°C to 45.50°C, respectively (minimum and maximum values over the whole study period).

**Figure 3:**
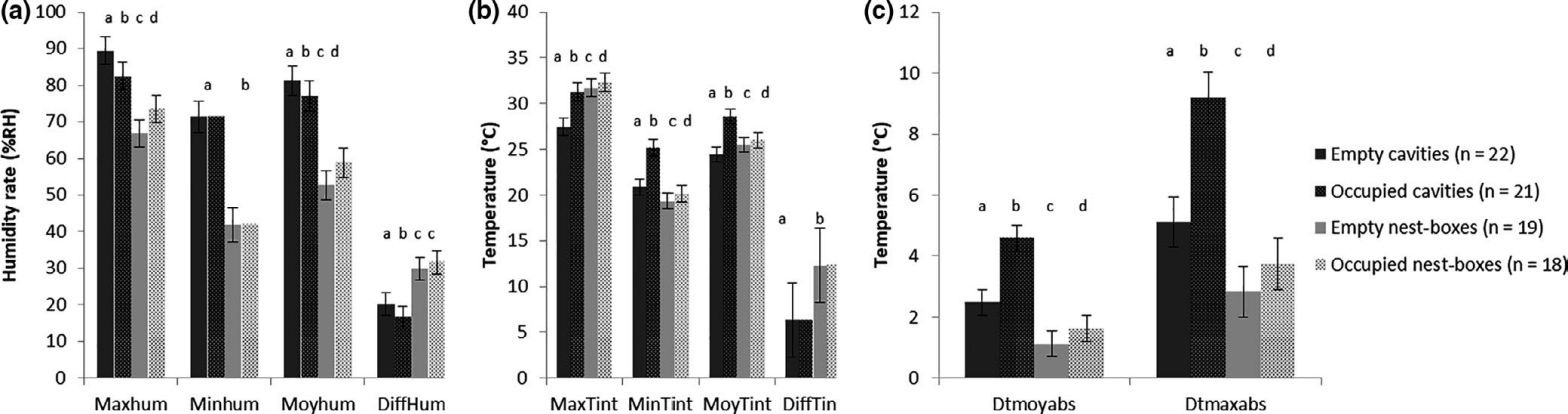
Comparison of the estimates of microclimate parameters (with their 95% confidence interval) between empty and occupied natural cavities and nest boxes in the Vallée des Baux (France). (A) maximum, minimum, average, and interior variation in humidity (Maxhum, Minhum, Moyhum, and DiffHum), (B) maximum, minimum, average, and interior variation in temperature (MaxTint, MinTint and MoyTint, DiffTint), and (C) maximum delta between interior and ambient temperature (Dtmaxabs) and average buffering capacity (Dtmoyabs). a, b, c & d: for each parameter, different letters indicate significant differences between the estimates of the corresponding nest type

Best models from model selection included the interaction of real occupation alone for all microclimate parameters except for the average interior temperature (MoyTint) maximum and average humidity rate (Maxhum and Moyhum) which also included developmental stage, and for interior temperature variation (DiffTint) and minimum humidity rate (Minhum) which only included nest type (Table S4). Empty nest boxes had a higher maximum (MaxTint: +4.26°C, *p < *.01), average (MoyTint: +1.09°C, *p = *.01), and lower minimum (MinTint: −1.51°C, *p < *.01) interior temperature, higher temperature variations (DiffTint: +94%, *p < *.01) and a lower average temperature buffering capacity (Dtmoyabs: −55%, *p < *.01) than empty cavities. Empty nest boxes also had a more variable (DiffHum: +47%, *p < *.01) and lower humidity rate (Moyhum: −28.58%RH, *p < *.01; Maxhum: −22.56%RH, *p < *.01; Minhum: −29.53%RH, *p < *.01) than empty cavities (Figure 3a). These differences were still significant and similar when birds were present in the nest during the monitoring period, except for average interior temperature, which tended to be higher in occupied cavities than in occupied nest boxes (MoyTint:+2.44°C, *p < *.01) (Figure 3b) (Table S5).

Maximum, average, and minimum interior temperature were higher in occupied nests than in empty nests for both nest types (Nest boxes: MaxTint: +0.6°C, *p = *.06; MoyTint: +0.45°C, *p* = .02; MinTint: +0.77°C, *p < *.01/ Natural cavities: MaxTint: +3.82°C, *p < *.01; MoyTint: +4.13°C, *p < *.01; MinTint: +4.30°C, *p < *.01). Similarly, occupation increased temperature buffering capacity in nests of both types (Nest boxes: Dtmoyabs: +46%, *p < *.01; Dtmaxabs: +31%, *p* < .01;/ Natural cavities: Dtmoyabs: +85%, *p < *.01; Dtmaxabs: +80%, *p < *.01) (Figure 3c). However, occupied nest boxes tended to be more humid than empty ones (Moyhum: +6.26%RH, *p < *.01; Maxhum: +6.83%RH, *p < *.01), while occupied cavities were significantly drier than empty cavities (Moyhum: −3.92%RH, *p < *.01; Maxhum: −6.48% RH, *p < *.01). Humidity rates were more stable in occupied natural cavities than in empty cavities (DiffHum: −19%, *p = *.02) but they had similar daily fluctuations in empty and occupied nest boxes. Average interior temperature decreased (*β* = −0.02, *p = *.03) while maximum and average interior humidity rates increased with increasing developmental stage of the clutch (Maxhum: *β* = 0.15; *p < *.01, Moyhum: *β* = 0.13; *p < *.01) (Table S5).

### 4.5 Effect of microclimate on occupation probability and breeding success

**Figure 4:**
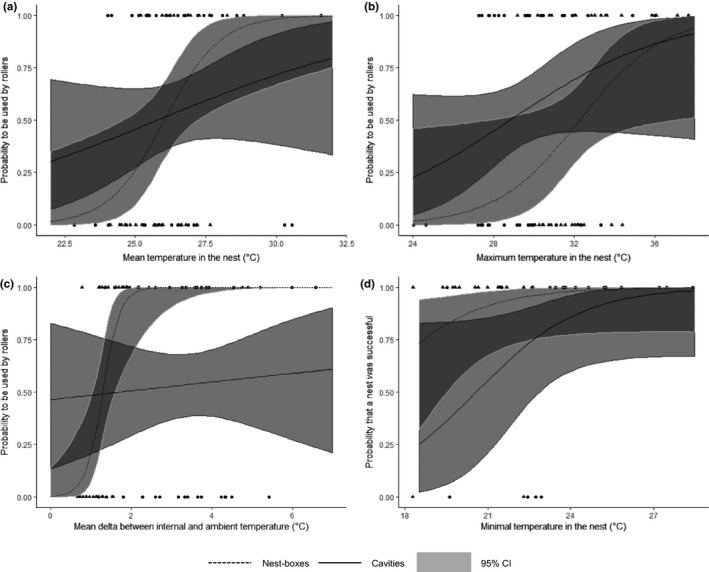
Correlation of different microclimate variables with occupation probability by the European roller (a: MOYTint, b: MAXTint, and c: DTMOYABS) and with the probability that a roller nest was successful (d: MINTint) for nest boxes (▲: dotted regression lines) and cavities (●:solid regression lines) in the Vallée des Baux (France). The 95% confidence intervals of regression lines are shown in gray, dark gray indicates overlaps between the intervals of the two nest types

European roller occupation probability increased slightly with average interior temperature (MOYTint) in nest boxes (*p = *.08) but not in natural cavities (Figure 4a). It increased marginally with maximum interior temperature for both nest types (MAXTint *p = *.10) (Figure 4b) but significantly with buffering capacity of the nest (DTMAXabs *p = *.05 and DTMOYabs *p < *.01) for nest boxes only (Figure 4c) (Table S6).

We found no effect of microclimate on clutch size, number of fledglings or fledging success per egg neither with nest type as an interaction factor (Table S6). However, in natural cavities, most microclimate variables were correlated with the probability that a nest was successful. This probability increased significantly with humidity rates (MOYhum *p = *.02, MINhum *p = *.02 and MAXhum *p = *.03), slightly with average buffering capacity (DTMOYabs *p = *.09), and minimum interior temperature (MINTint *p = *.08) (Figure 4d), whereas it decreased marginally when interior temperature and humidity variation increased (DIFFTint *p = *.06 and DIFFhum *p = *.07, respectively) (Table S6).

## 5 DISCUSSION

Our results showed a strong contrast between nest types in terms of microclimatic conditions. Natural cavities had higher humidity on average and buffered ambient temperature generally more than nest boxes. Our results showed an impact of different microclimate parameters on occupation probability by European rollers, which selected the warmest and better buffered artificial nests. The higher occupation rates of nest boxes and similar egg‐laying dates showed that artificial devices were preferred or equally selected by European rollers compared with natural cavities, but we did not find any significant differences in European roller breeding success between the two nest types. This suggests that nest boxes are not ecological traps for European rollers in this study area.

### 5.1 Microclimate differs significantly between nest types

Nest orientation was correlated with several microclimate parameters, confirming other studies that showed that this nest characteristic is an important microclimatic driver in both cavities and nest boxes (Ardia et al., 2006). North‐facing nests are typically less exposed to sunlight—and hence to heat—compared with other orientations in the northern hemisphere, and thus tend to have lower interior maximum temperatures (Wiebe, 2001) and higher humidity rates (Di Maggio et al., 2015).

Because of the deployment strategy of the nest boxes, they differ significantly from natural cavities in terms of nest height and orientation, with cavities being averagely 1.63m higher and nest boxes being oriented mostly to the West or to the South. These differences could act as confounding factors for potential microclimate differences between nest types. However, nest height was not correlated to any microclimate parameter. Furthermore, even after controlling for nest orientation, we found strong microclimate differences between nest types, both for temperature and humidity rate. The results confirm the poor temperature and humidity buffering capacity of plywood nest boxes compared to natural tree cavities, as tree trunks provide much better climatic insulation compared to the thin walls of nest boxes (Rowland et al., 2017). Similar results were found in another study in the European roller between wooden nest boxes and natural or seminatural cavities in sand cliffs, bridges, and buildings (Amat‐Valero et al., 2014; Catry et al., 2015). Combined with our results, this demonstrates that wooden nest boxes are not reproducing the microclimate of any of the most common natural or seminatural nest types of the European roller throughout its breeding range (Cramp, 1985), and thus cannot be treated as an equivalent substitute of natural nesting sites.

The presence of birds in the nest (both European roller and common starling) during sensor deployment affected microclimate significantly, increasing interior temperature and temperature buffering capacity in both natural cavities and nest boxes. However, we found similar differences between nest types in both empty and occupied nests. These results are in line with previous studies that found that even a much smaller bird, great tits *Parus major,* increased interior temperature and temperature buffering capacity in nest boxes (Maziarz & Wesołowski, 2013; Veľký et al., 2010) as well as marsh tits *Poecile palustris* in natural cavities (Maziarz, 2019). Similarly to Maziarz (2019), and Maziarz and Wesołowski (2013), we found that humidity rates were lower in occupied compared with empty cavities. However, we found a reverse relationship in nest boxes. We also found an effect of developmental stage of the clutch on three microclimate parameters, but the relationship was reverse to the one found by Maziarz (2019) in the marsh tit: average interior temperature decreased with the growth of the chicks, while maximum and average interior humidity rates increased. These results could be explained by the huge difference of microclimate between nest types: The presence of birds in the nest could buffer temperature and humidity levels to a medium level, in between the dry empty nest boxes and the humid empty cavities. The effect of the presence of birds in the nest on microclimate underlines the greater energetic cost for birds using nest boxes which showed greater microclimatic variations compared with natural cavities in our study area (Maziarz, 2019).

Nesting sites occupied by European rollers could also contain old nest material from competitor species or layers of defecations of chicks of European rollers, when occupied during previous years, which might modify the internal microclimate (see e.g., Blem and Blem (1994), Maziarz and Wesołowski (2013)). However, nest boxes were emptied every year after the breeding season in our study site and previous studies have shown that nest material in natural cavities tends to disappear very quickly (Wesołowski, 2000). Nests constructed by competitor species such as the common starling, within cavities before the settlement of European rollers could also modify the volume—and hence the microclimate—of natural cavities. However, we found no effect of cavity length—the only measured cavity characteristic possibly affected by nest volume in our study—on any microclimate parameter in our study, thus suggesting that the effect of nest material on internal microclimate might be small for cavities available for European rollers in our study area. This hypothesis should be explored in future studies. In our study, the measured microclimate parameters were not correlated to Julian calendar date. Therefore, we can consider that the measured microclimate in each nest is representative of the whole breeding period of European rollers, when corrected for the presence of birds in the nest.

### 5.2 European rollers prefer artificial over natural nests despite more stressful microclimatic conditions

We found that nest boxes had higher occupation rates compared with natural cavities, suggesting a strong preference for this nest type. The observed preference for nest boxes could be due to their higher visibility compared with natural nests, which could ease their detection by European rollers. As such, Rodríguez‐Ruiz et al. (2011) found that more visible nest boxes were preferred by European rollers compared with more concealed ones. This hypothesis could be explored in the future in both natural and artificial cavities in the study area. This observed preference could also be due to a better protection against predators in nest boxes than in natural cavities, a benefit which has been identified for other species, for example, in the Madeiran storm petrel *Oceanodroma castro* (Bolton et al., 2004). In our study, we found a slightly higher frequency of predation (excluding unknown failure events) in natural nests compared with nest boxes (*n* = 6 and *n* = 4, respectively, 8.7% and 5.7% of recorded breeding attempts in respective nest type in our study area between 2016 and 2019). Although this difference was not significant on our study area, the reduced intensity in the monitoring of cavities compared to nest boxes (see Section 2) could mean that we could have not detected some predation events at the early stage of the reproduction (e.g., during egg laying or incubation period). The real frequency of predation events in natural cavities might hence be higher than the one we observed. Long‐term breeding monitoring and more intensive surveys of natural cavities would give more insights to validate this hypothesis in the future.

Observed predation rates were, however, extremely low in both nest types and support the findings of Parejo and Avilès (2011) showing that European rollers are able to assess predation risk in nest site selection, similarly to several cavity‐nesting birds (Fontaine & Martin, 2006; Mönkkönen et al., 2009; Wesołowski, 2002). It would thus be possible that preference for safe nesting sites could lead European rollers to select for less suitable thermal or humidity conditions in order to reduce the nest predation risk, as suggested in the great tit (Maziarz et al., 2016). However, this hypothesis was not supported by our findings for the European roller: Of the available nest boxes, European rollers selected the warmest ones, with high maximal (MAXTint) and average interior temperature (MOYTint). Warmer nests may be especially attractive to cavity‐nesting altricial birds, as they could reduce energetic costs and improve hatching success, especially at the beginning of the breeding season, when minimal ambient temperatures are still low (Reid et al., 2000). Early‐season microclimatic conditions in nest boxes could lead European rollers to select these warmer artificial nests at the risk of exposing their chicks to more stressful extremely high temperatures in the following weeks (Catry et al., 2015). However, when selecting a nest box, European rollers also tended to occupy the nests with better temperature buffering capacity (DTMAXabs and DTMOYabs). This result suggests that European rollers could be able to avoid the most stressful microclimatic conditions among the available nest boxes, independently from nest predation risk.

### 5.3 European roller breeding success does not differ between nest types

Contrary to our prediction, we found no significant difference in breeding success parameters between artificial and natural nests.

The probability of a breeding pair bringing at least one chick to fledging in occupied natural nests was significantly positively correlated with humidity rates (MAXhum, MINhum, MOYhum), marginally negatively correlated with humidity and temperature variation (DIFFHum, DIFFTint) and marginally positively correlated with buffering capacity (DTMOYabs) and minimum temperature (MINTint) in the nest. These results confirmed that dry and poorly buffered microclimatic conditions negatively impact fitness. However, all these parameters were significantly more favorable in natural cavities than in nest boxes. Thus, we would have expected higher breeding success in natural nests, as demonstrated in some other bird species, for example, in the African penguin *Speniscus demersus* (Lei et al., 2014) or in the lesser kestrel *Falco naumanii* (Catry et al., 2015).

The absence of significant disparities between nest types could be explained by European roller occupation strategy. As European rollers avoided the nest boxes with the least favorable microclimates (see Section 5.2), the induced stress in the selected artificial nests may have been sufficiently reduced as to not significantly impact the individual fitness. The positive impact of this strategy may have been additionally strengthened by the particular heat tolerance of European rollers compared to other bird species. Catry et al. (2015) found that in nest boxes in Portugal European roller chicks suffer much less from extreme heat conditions than those of the Lesser kestrel. It is possible that European rollers have sufficient plasticity to tolerate a certain amount of microclimatic stress, for instance through adaptive parental behavior. Furthermore, Catry et al. (2015) showed that European roller chicks could survive interior maximum temperatures of more than 50°C, which largely exceeds maximum interior temperatures recorded in our study (43.5°C), despite similar heat waves with days exceeding 37°C of maximum exterior temperature in June, July, and August 2017 (www.meteofrance.fr). Nest boxes directly exposed to sunlight can have interior maximum temperature that largely exceeds ambient temperature (Ellis, 2016). All the nest boxes in our study area are placed on tree trunks and therefore benefit from the shade of branches and foliage, which is not the case in nest boxes placed on poles and buildings in the study area described by Catry et al. (2015). This could explain the lower maximum temperatures found in the nest boxes on our study area.

In southern Spain, Valera et al. (2019) also did not find breeding success disparities in the European roller between wooden nest boxes and natural and seminatural cavities, despite microclimatic disparities between nest types similar than found in our study (Amat‐Valero et al., 2014). However, to our knowledge, they did not explore the link between the two. Microclimate disparities between nest types could also influence parasite presence and abundance, as demonstrated in Spain in an ectoparasite of the European roller, *Carnus hemapterus* (Amat‐Valero et al., 2013, 2014), which could impact fitness, nest site selection, and breeding success (Loye & Carroll, 1998) and should be explored in the future in our study area.

Overall, our results found that European roller fitness was not negatively impacted in the nest boxes they selected, at least in terms of breeding success. However, the least favorable microclimate in nest boxes probably increases energy expenditure of European rollers, as also suggested in the great tit (Maziarz, 2019), which could have other fitness consequences, and affect traits such as chick growth (Andreasson et al., 2018), body condition (Catry et al., 2011), or postfledging survival (Greño et al., 2008), which would be valuable to explore in future studies. Nonetheless, according to our conceptual framework, in our study area, nest boxes for European rollers must be considered successful conservation measures. By avoiding the nest boxes with the less suitable microclimate, it is possible that European rollers avoid potential ecological traps, contrary to our initial hypothesis. It would be necessary to test this hypothesis in the future by comparing the influence of microclimate on occupancy in the European roller between artificial and natural nests, over a climatic and geographical gradient.

### 5.4 Recommendations for nestboxes placement

In southern France, European roller breeding density is limited by nest availability (Finch et al., 2019). In our study area, nest boxes were more attractive than natural cavities and did not deter breeding success of European rollers. Therefore, providing nest boxes as additional available nesting places could be beneficial to conservation of the species.

While we found that European rollers appear to avoid the least favorable nest boxes among those available, this choice was only possible because these were located in different settings, under tree canopy, which created a variety of microclimates. This might not always be the case: for instance, in open habitats, nest boxes are usually placed on electricity poles (Aleman & Laurens, 2013; Monti et al., 2019) and hence directly exposed to sunlight. To avoid extreme microclimatic conditions in nest boxes for cavity breeders such as European rollers, we recommend the following:

1. In Mediterranean and tropical regions, place nest boxes in shaded conditions and oriented northwest to northeast (southwest to southeast in Southern hemisphere). This reduces both maximum temperatures and temperature variation (see also Catry et al., 2011). However, in colder regions of the northern hemisphere, nest boxes should be south‐facing in order to favor early‐breeding species, especially at the beginning of the breeding season (Ardia et al., 2006; Dawson et al., 2005). Similarly, we recommend avoiding placing nest boxes on electricity or telephone poles, as these provide no shade. This is especially important in warm regions, where birds can suffer from overheating, but cold nights can also affect bird survival in every part of the world in such poorly buffered microclimatic conditions.
2. Build nest boxes using materials with good insulation properties, such as carved logs or tree trunks (Griffiths et al., 2018), as it will reduce maximal and increase minimal temperature in the nest, as well as daily fluctuations, and increase humidity rate, and hence improve attractivity of the nest and the probability of laid eggs to produce fledglings.

These measures should reduce the probability of nest boxes creating deleterious microclimatic conditions for their occupants. Our findings also confirm previous studies showing that nest boxes poorly replicate the microclimate of tree cavities. Therefore, we highlight the need to focus on protecting cavity trees, while treating nest boxes as temporary solution in species conservation, whose significant installation and maintenance costs could be difficult to sustain in the long term (Goldingay et al., 2018; Lindenmayer et al., 2009, 2017).

## 6 CONCLUSIONS

Our findings demonstrated that for the European roller in our study area, nest boxes were preferred over natural cavities and that breeding success between the two nest types was not significantly different. The European rollers appeared to avoid the least favorable nest boxes, and while the microclimatic conditions of artificial nests were more variable, this neither deterred occupation nor translated into lower breeding success. However, the large microclimate differences observed between nest types might affect energy expenditure of birds and could hence deter other bird fitness parameters or proxies which would be valuable to explore in future studies.

Using the framework of the ecological trap hypothesis enabled us to compare preference and a fitness parameter (here fecundity) between a natural habitat—tree cavities—and nest boxes in order to evaluate the effectiveness of the latter for the conservation of a secondary cavity breeder. This framework is potentially applicable to any species in which some populations use restored or artificial habitats as well as unmodified or natural habitats at a given stage of their life history.

However, this framework focuses solely on the ecology of the target species, while evaluating habitat restoration programs should take other considerations into account. A more holistic approach could include an evaluation of (a) the durability or resilience of the restored or artificial habitat in comparison with the natural or unmodified habitat, (b) the social and economic costs of restoration compared to conservation outcomes, and (c) ecosystem functionality, that is, placing restored habitats and target species in a broader ecological network, taking into account the relationship between landscape elements and their structuration, and the fitness of the target species, which would enable finer‐scale recommendations.

## CONFLICT OF INTEREST

None declared.

## AUTHOR CONTRIBUTION

**Timothée Schwartz:** Conceptualization (equal); Data curation (equal); Formal analysis (lead); Funding acquisition (equal); Investigation (equal); Methodology (equal); Project administration (lead); Writing‐original draft (lead); Writing‐review & editing (lead). **Arnaud Genouville:** Data curation (equal); Formal analysis (equal); Investigation (equal); Methodology (equal); Writing‐original draft (equal); Writing‐review & editing (supporting). **Aurélien Besnard:** Conceptualization (equal); Formal analysis (supporting); Funding acquisition (equal); Methodology (equal); Project administration (supporting); Supervision (lead); Writing‐original draft (equal); Writing‐review & editing (equal).

## Supporting information

Appendix S1

## Data Availability

Data files used to perform the analysis are available from the Dryad Digital Repository: https://doi.org/10.5061/dryad.xksn02vdg.
