# Supplementary material for: Increased microclimatic variation in artificial nests does not create ecological traps for a secondary cavity breeder, the European roller
Source: Ecol Evol. 2020 Nov 18;10(24):13649–63. doi: 10.1002/ece3.6871 (PMC7771184; doi:10.1002/ece3.6871)
Supplement: Supplementary file 1 — Appendix S1 [file ECE3-10-13649-s001.docx]

**Appendix S1 - Supplementary figures and tables**


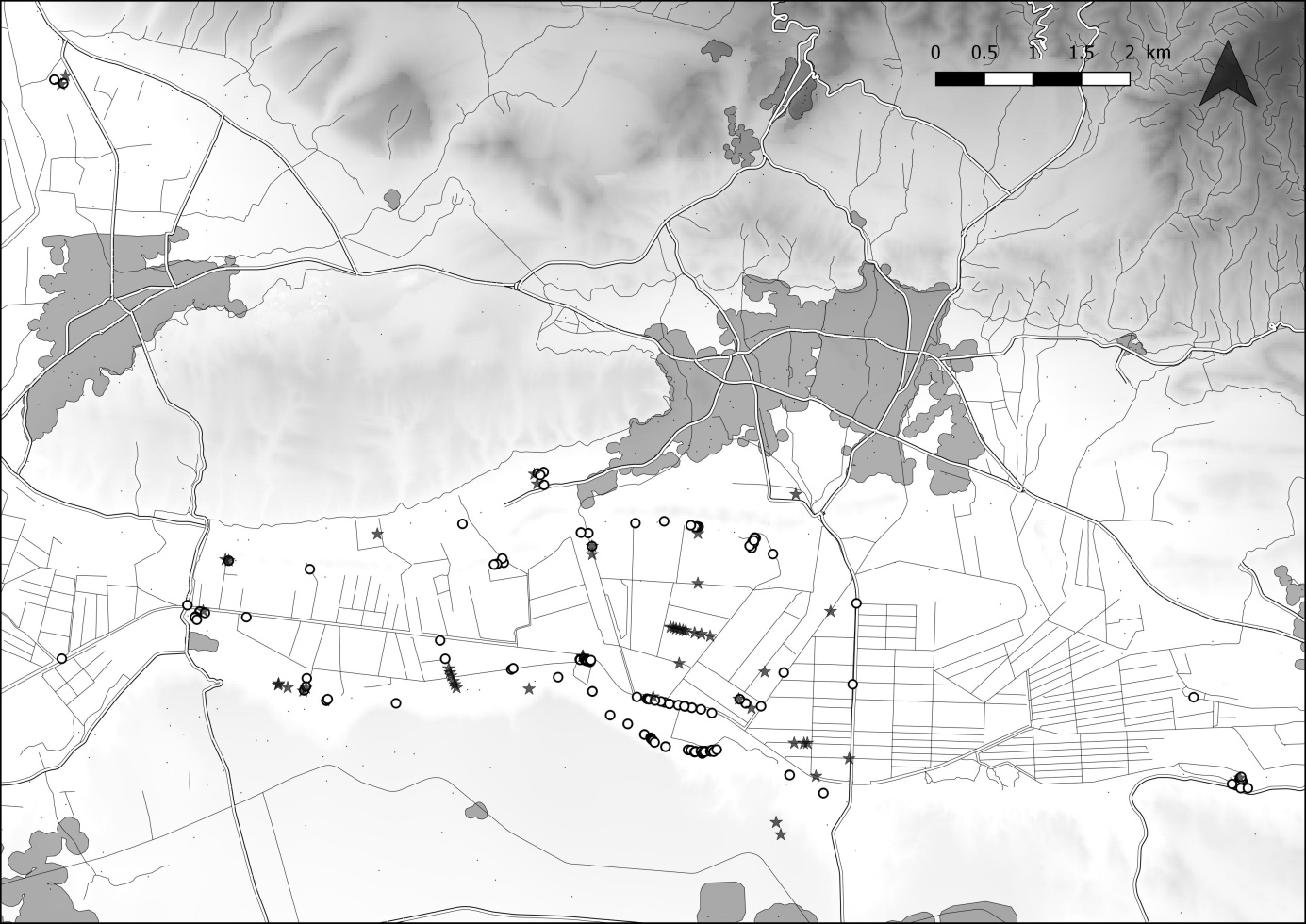


Figure S1: Map of the study area and distribution of natural cavities (rounds) and nest-boxes (stars) available for the reproduction of European rollers between 2016 and 2019.


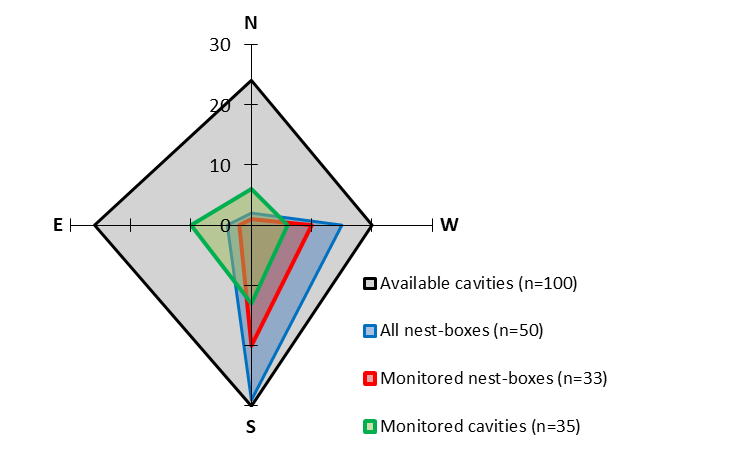


Figure S2: Orientation of cavities (n = 35) and nest-boxes (n = 33) monitored for microclimate, and of available cavities (n = 100) and nest-boxes (n = 50) for European roller reproduction in the Vallée des Baux (France) in 2017 (N: north, W: west, S: south, E: east).


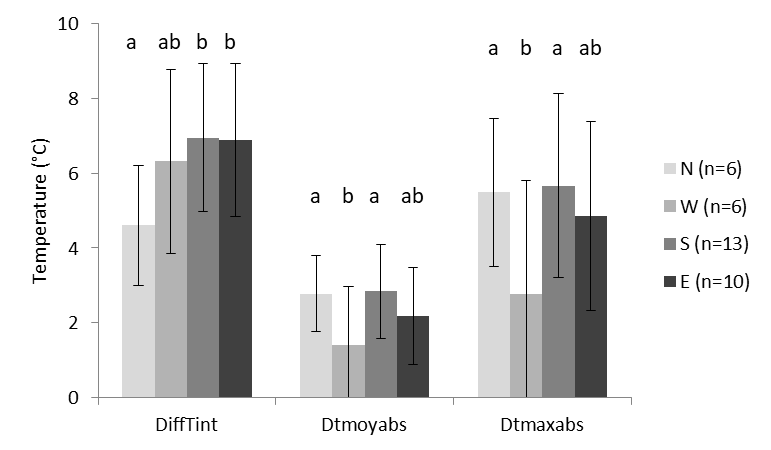

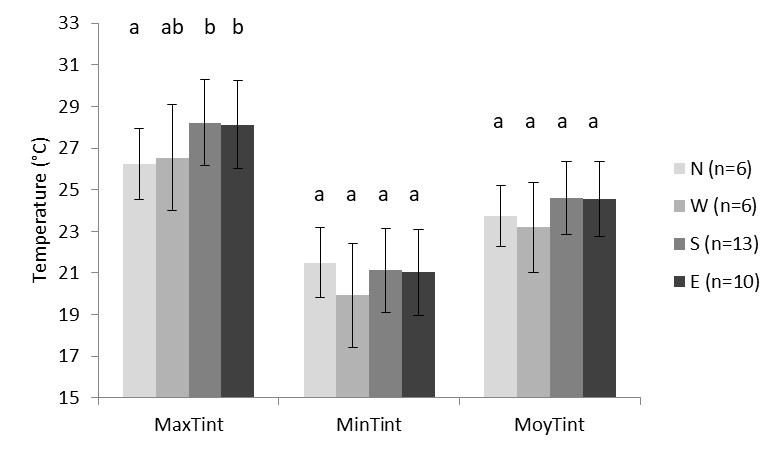

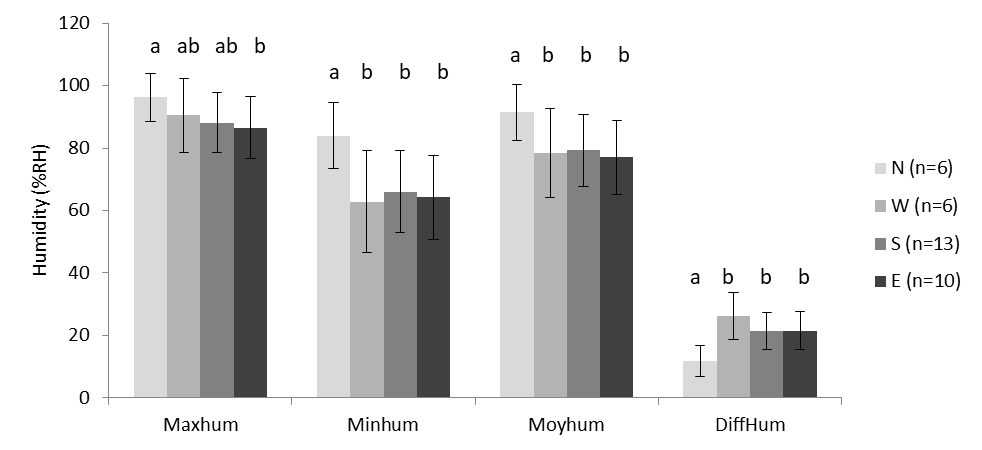


(B)

(A)

(C)

Figure S3: Estimates of microclimate parameters with their 95% confidence interval for different orientations (N: north, W: west, S: south, E: east) in natural cavities in the Vallée des Baux (France): (A) internal temperature variation (DiffTint), average buffering capacity (Dtmoyabs) and maximum delta between interior and ambient temperature (Dtmaxabs), (B) maximum, minimum and average interior temperature (MaxTint, MinTint and MoyTint respectively) and (C) maximum, minimum, average and interior variation in humidity (Maxhum, Minhum, Moyhum and DiffHum). a, b, c & d: for each parameter, different letters indicate significant differences between the estimates of the corresponding nest type.

Fitted line (OccR = 1)

Fitted line (OccR = 0)

95% Confidence interval (OccR = 1)

95% Confidence interval (OccR = 0)


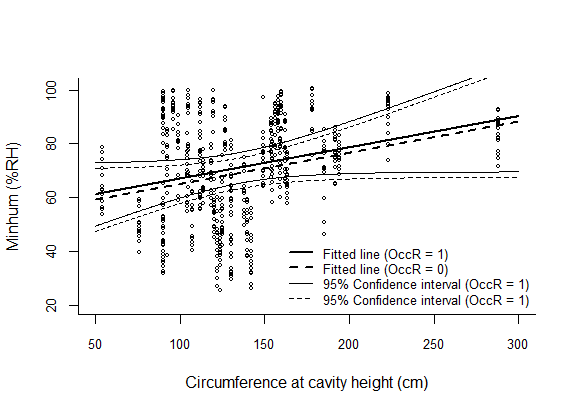


(C)


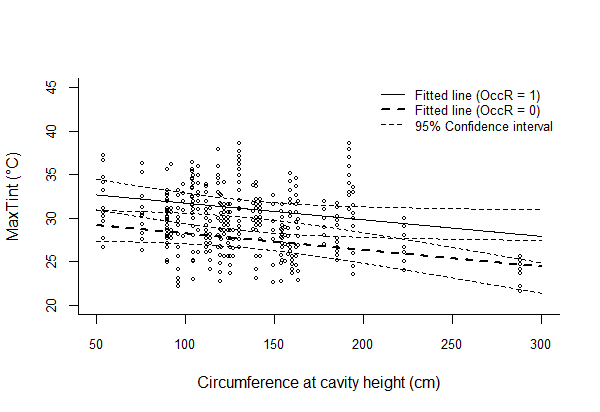


(A)


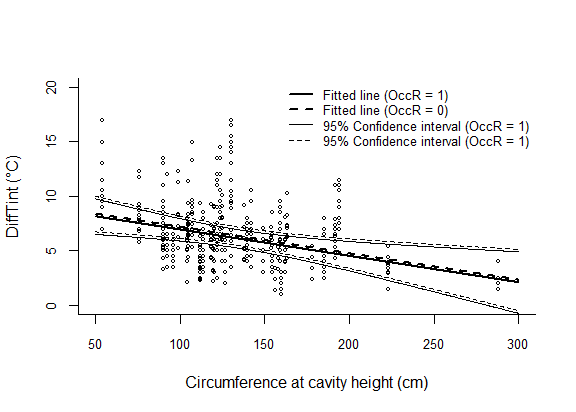


(B)

Figure S4: Correlation between circumference at cavity height and microclimate parameters in cavities available for European roller reproduction in the Vallée des Baux (France) with the additive effect of the occupation of nest during microclimate measurements (OccR): (A) maximum internal temperature (MaxTint); (B) internal temperature variation (DiffTint); (C) minimum humidity rate (Minhum). Dotted lines represent the 95% CI of the regression line.

Table S1: Date of the start of microclimate measurements, occupation status by European rollers and first egg-laying date in occupied nests, for two different types of nesting sites (41 natural cavities and 33 nest-boxes) monitored in the Vallée des Baux (France) in 2017.

| ID | Start of measurements (Julian calendar date) | First egg-laying date (Julian calendar) | Occupation by rollers | Type |
| --- | --- | --- | --- | --- |
| Cav115.1 | 193 | NA | 1 | Cavity |
| Cav116.4 | 191 | NA | 1 | Cavity |
| Cav14 | 158 | 156 | 1 | Cavity |
| Cav154 | 167 | 150 | 1 | Cavity |
| Cav155 | 182 | 153 | 1 | Cavity |
| Cav156 | 216 | 153 | 1 | Cavity |
| Cav157 | 201 | 151 | 1 | Cavity |
| Cav2.1 | 157 | 151 | 1 | Cavity |
| Cav23.1 | 209 | 145 | 1 | Cavity |
| Cav30 | 163 | 149 | 1 | Cavity |
| Cav32 | 209 | 150 | 1 | Cavity |
| Cav35.1 | 173 | 141 | 1 | Cavity |
| Cav38.2 | 170 | 140 | 1 | Cavity |
| Cav48.3 | 170 | 140 | 1 | Cavity |
| Cav50.1 | 170 | 140 | 1 | Cavity |
| Cav52 | 171 | 143 | 1 | Cavity |
| Cav55 | 158 | 136 | 1 | Cavity |
| Cav63 | 188 | 149 | 1 | Cavity |
| Cav67 | 160 | 142 | 1 | Cavity |
| Cav75 | 166 | 150 | 1 | Cavity |
| Cav78 | 185 | 141 | 1 | Cavity |
| Cav88.2 | 180 | 152 | 1 | Cavity |
| Cav106 | 204 | NA | 0 | Cavity |
| Cav19.2 | 202 | NA | 0 | Cavity |
| Cav2.2 | 180 | NA | 0 | Cavity |
| Cav23.2 | 194 | NA | 0 | Cavity |
| Cav3 | 158 | NA | 0 | Cavity |
| Cav31.1 | 203 | NA | 0 | Cavity |
| Cav33 | 202 | NA | 0 | Cavity |
| Cav48.1 | 170 | NA | 0 | Cavity |
| Cav50.2 | 170 | NA | 0 | Cavity |
| Cav54 | 201 | NA | 0 | Cavity |
| Cav57 | 195 | NA | 0 | Cavity |
| Cav62 | 201 | NA | 0 | Cavity |
| Cav64 | 188 | NA | 0 | Cavity |
| Cav77 | 166 | NA | 0 | Cavity |
| Cav79 | 168 | NA | 0 | Cavity |
| Cav8 | 194 | NA | 0 | Cavity |
| Cav80 | 209 | NA | 0 | Cavity |
| Cav96.2 | 202 | NA | 0 | Cavity |
| CavEsclade 1 | 207 | NA | 0 | Cavity |
| 3 | 157 | 144 | 1 | Nest-box |
| 5 | 158 | 144 | 1 | Nest-box |
| 13 | 158 | 139 | 1 | Nest-box |
| 17 | 188 | 148 | 1 | Nest-box |
| 20 | 196 | 154 | 1 | Nest-box |
| 30 | 196 | 142 | 1 | Nest-box |
| 35 | 193 | 140 | 1 | Nest-box |
| 37 | 163 | 152 | 1 | Nest-box |
| 55 | 167 | 144 | 1 | Nest-box |
| 57 | 167 | 150 | 1 | Nest-box |
| 69 | 189 | 155 | 1 | Nest-box |
| 71 | 199 | 176 | 1 | Nest-box |
| 72 | 163 | 147 | 1 | Nest-box |
| 74 | 163 | 158 | 1 | Nest-box |
| 82 | 158 | 150 | 1 | Nest-box |
| 85 | 207 | 147 | 1 | Nest-box |
| 86 | 158 | 165 | 1 | Nest-box |
| 1 | 166 | NA | 0 | Nest-box |
| 4 | 158 | NA | 0 | Nest-box |
| 9 | 163 | NA | 0 | Nest-box |
| 19 | 193 | NA | 0 | Nest-box |
| 36 | 163 | NA | 0 | Nest-box |
| 39 | 182 | NA | 0 | Nest-box |
| 45 | 181 | NA | 0 | Nest-box |
| 53 | 181 | NA | 0 | Nest-box |
| 56 | 182 | NA | 0 | Nest-box |
| 59 | 181 | NA | 0 | Nest-box |
| 70 | 193 | NA | 0 | Nest-box |
| 75 | 193 | NA | 0 | Nest-box |
| 77 | 186 | NA | 0 | Nest-box |
| 80 | 181 | NA | 0 | Nest-box |
| 81 | 200 | NA | 0 | Nest-box |
| 84 | 200 | NA | 0 | Nest-box |

Table S2: Monthly mean of the daily average, daily minimum and daily maximum temperatures (°C) recorded in Salon de Provence (43.60°N, 5.11°E) in May, June and July from 2016 to 2019 (www.meteofrance.fr).

|  | 2016 | | |  | 2017 | | |  | 2018 | | |  | 2019 | | |
| --- | --- | --- | --- | --- | --- | --- | --- | --- | --- | --- | --- | --- | --- | --- | --- |
|  | May | June | July |  | May | June | July |  | May | June | July |  | May | June | July |
| Minimum T (°C) | 10.7 | 15.7 | 19.0 |  | 11.5 | 17.5 | 18.1 |  | 12.8 | 16.6 | 18.8 |  | 11.4 | 15.2 | 19.2 |
| Maximum T (°C) | 22.0 | 28.4 | 31.9 |  | 24.1 | 29.3 | 29.9 |  | 23.1 | 27.7 | 32.0 |  | 22.3 | 28.5 | 31.7 |
| Average T (°C) | 16.4 | 22.1 | 25.4 |  | 17.8 | 23.4 | 24.0 |  | 17.9 | 22.2 | 25.4 |  | 16.9 | 21.9 | 25.5 |

Table S3.a: Correlations between nest characteristics and microclimate variables in nest-boxes and natural cavities available for European roller reproduction in the Vallée des Baux (France). CH: cavity height. CBH: circumference at breast height. CCH: circumference at cavity height. EH: entrance height. EW: entrance width. CL: cavity length. CD: cavity depth (=horizontal length). MaxTint: daily maximal temperature in the nest. MinTint: daily minimal temperature in the nest. MoyTint: daily mean temperature in the nest. DiffTint: difference between daily maximal and minimal temperature in the nest. Dtmaxabs: absolute value of the daily maximum delta between interior and exterior temperature. Dtmoyabs: mean over a day of the absolute delta between interior and exterior temperature. Maxhum: daily maximum humidity level in the nest. Minhum: daily minimum humidity level in the nest. Moyhum: daily mean humidity level in the nest. DiffHum: difference between daily maximal and minimal humidity level in the nest. “β_x_”: estimate of the slope of the correlation. “SE”: standard error of the estimate. “t”: t-value for the corresponding estimate. “*P”*: p-value. “n”: sample size. *P*-values lower than 0.10 are in bold.

|  | Both nest types (n = 63) | | | | | |  | Natural cavities only (n = 35) | | | | | | | | | | | | | | | | | | | | | | |
| --- | --- | --- | --- | --- | --- | --- | --- | --- | --- | --- | --- | --- | --- | --- | --- | --- | --- | --- | --- | --- | --- | --- | --- | --- | --- | --- | --- | --- | --- | --- |
|  | CH | | | | | |  | CBH | | |  | CCH | | |  | EH | | |  | EW | | |  | CL | | |  | CD | | |
| Response variables | Natural cavities  (n = 35) | | | Nest-boxes  (n = 28) | | |  |  |  |  |  |  |  |  |  |  |  |  |  |  |  |  |  |  |  |  |  |  |  |  |
|  | βx (SE) | t | *P* | βx (SE) | t | *P* |  | βx (SE) | t | *P* |  | βx (SE) | t | *P* |  | βx (SE) | t | *P* |  | βx (SE) | t | *P* |  | βx (SE) | t | *P* |  | βx (SE) | t | *P* |
| MaxTint | -0.04 (0.13) | -0.34 | 0.74 | 0.57 (0.62) | 0.92 | 0.36 |  | **-0.85 (0.42)** | **-2.02** | **0.05** |  | **-0.80 (0.37)** | **-2.13** | **0.04** |  | 0.02 (0.39) | 0.06 | 0.96 |  | -0.24 (0.39) | -0.62 | 0.54 |  | 0.56 (0.40) | 1.40 | 0.17 |  | 0.57 (0.44) | 1.29 | 0.21 |
| MinTint | -0.01 (0.11) | -0.14 | 0.89 | -0.01 (0.51) | -0.03 | 0.98 |  | -0.34 (0.34) | -1.01 | 0.32 |  | 0.23 (0.36) | 0.63 | 0.53 |  | 0.39 (0.36) | 1.11 | 0.28 |  | -0.13 (0.36) | -0.36 | 0.72 |  | 0.48 (0.37) | 1.28 | 0.21 |  | 0.28 (0.35) | 0.81 | 0.42 |
| MoyTint | 0.01 (0.09) | 0.16 | 0.88 | 0.17 (0.46) | 0.38 | 0.71 |  | **-0.60 (0.33)** | **-1.84** | **0.08** |  | -0.28 (0.32) | -0.85 | 0.40 |  | 0.21 (0.32) | 0.66 | 0.51 |  | -0.16 (0.32) | -0.52 | 0.61 |  | 0.46 (0.33) | 1.39 | 0.17 |  | 0.44 (0.34) | 1.28 | 0.21 |
| DiffTint | -0.02 (0.13) | -0.19 | 0.85 | 0.59 (0.64) | 0.93 | 0.36 |  | -0.46 (0.41) | -1.14 | 0.26 |  | **-1.02 (0.35)** | **-2.90** | **0.01** |  | -0.37 (0.38) | -0.99 | 0.33 |  | -0.11 (0.38) | -0.29 | 0.77 |  | 0.10 (0.38) | 0.28 | 0.78 |  | 0.27 (0.42) | 0.66 | 0.51 |
| Dtmoyabs | 0.04 (0.06) | 0.59 | 0.55 | -0.03 (0.32) | -0.12 | 0.91 |  | -0.05 (0.25) | -0.19 | 0.85 |  | 0.36 (0.23) | 1.56 | 0.13 |  | -0.01 (0.23) | -0.05 | 0.96 |  | -0.27 (0.23) | -1.19 | 0.24 |  | 0.23 (0.24) | 0.96 | 0.35 |  | 0.22 (0.26) | 0.84 | 0.41 |
| Dtmaxabs | 0.02 (0.13) | 0.20 | 0.85 | 0.12 (0.61) | 0.21 | 0.84 |  | -0.26 (0.52) | -0.51 | 0.61 |  | 0.59 (0.45) | 1.30 | 0.20 |  | 0.10 (0.46) | 0.24 | 0.82 |  | -0.26 (0.46) | -0.57 | 0.57 |  | 0.64 (0.47) | 1.35 | 0.19 |  | 0.52 (0.52) | 0.99 | 0.33 |
| Maxhum | -0.87 (0.58) | -1.48 | 0.14 | 1.18 (2.74) | 0.43 | 0.67 |  | 1.99 (1.87) | 1.07 | 0.29 |  | 2.91 (1.79) | 1.63 | 0.11 |  | -1.45 (1.81) | -0.80 | 0.43 |  | -1.74 (1.81) | -0.97 | 0.34 |  | -0.46 (1.86) | -0.25 | 0.80 |  | -2.07 (1.90) | -1.09 | 0.28 |
| Minhum | -1.12 (0.83) | -1.34 | 0.18 | -1.04 (3.92) | -0.27 | 0.79 |  | 2.50 (2.73) | 0.92 | 0.37 |  | **4.92 (2.61)** | **1.89** | **0.07** |  | 0.00 (2.69) | 0.00 | 1.00 |  | -2.28 (2.67) | -0.85 | 0.40 |  | 1.48 (2.71) | 0.55 | 0.59 |  | -1.02 (2.80) | -0.36 | 0.72 |
| Moyhum | -0.98 (0.70) | -1.39 | 0.17 | 0.10 (3.30) | 0.03 | 0.98 |  | 2.06 (2.30) | 0.90 | 0.38 |  | **3.71 (2.19)** | **1.69** | **0.10** |  | -1.01 (2.24) | -0.45 | 0.65 |  | -2.30 (2.22) | -1.04 | 0.31 |  | 0.16 (2.27) | 0.07 | 0.94 |  | -1.88 (2.35) | -0.80 | 0.43 |
| DiffHum | 0.24 (0.45) | 0.54 | 0.59 | 2.23 (2.11) | 1.06 | 0.29 |  | -0.61 (1.32) | -0.46 | 0.65 |  | -2.04 (1.27) | -1.60 | 0.12 |  | -1.40 (1.28) | -1.09 | 0.28 |  | 0.53 (1.30) | 0.41 | 0.69 |  | -1.72 (1.31) | -1.32 | 0.20 |  | -0.87 (1.34) | -0.65 | 0.52 |

Table S3.b: Comparison of the estimates of microclimate variables between the main orientations (N: north, W: west, S: south, E: east) of natural and artificial nests available for European roller reproduction in the Vallée des Baux (France). MaxTint: daily maximal temperature in the nest. MinTint: daily minimal temperature in the nest. MoyTint: daily mean temperature in the nest. DiffTint: difference between daily maximal and minimal temperature in the nest. Dtmaxabs: absolute value of the daily maximum delta between interior and exterior temperature. Dtmoyabs: mean over a day of the absolute delta between interior and exterior temperature. Maxhum: daily maximum humidity level in the nest. Minhum: daily minimum humidity level in the nest. Moyhum: daily mean humidity level in the nest. DiffHum: difference between daily maximal and minimal humidity level in the nest. “SE”: standard error of the estimate. “*P”*: p-value. “n”: sample size. *P*-values lower than 0.10 are in bold.

|  |  | Natural cavities (n = 35) | | | | | | | | | | | | | | | | | Nest-boxes (n = 30) | | | | | |
| --- | --- | --- | --- | --- | --- | --- | --- | --- | --- | --- | --- | --- | --- | --- | --- | --- | --- | --- | --- | --- | --- | --- | --- | --- |
| Response variables |  | N (n = 6) | | | | |  | E (n = 10) | | | |  | S (n = 13) | | |  | W (n = 6) | | S (n = 20) | | |  | W (n = 10) | |
|  |  | Estimate | SE | *P* | | |  | Estimate | SE | *P* | |  | Estimate | SE | *P* |  | Estimate | SE | Estimate | SE | *P* |  | Estimate | SE |
|  |  |  |  | S | E | W |  |  |  | S | W |  |  |  | W |  |  |  |  |  | W |  |  |  |
| MaxTint |  | 26.23 | 1.08 | **0.07** | **0.09** | 0.81 |  | 28.12 | 0.78 | 0.92 | 0.21 |  | 28.22 | 0.97 | 0.17 |  | 26.54 | 1.23 | 31.43 | 0.65 | 0.23 |  | 32.22 | 0.63 |
| MinTint |  | 21.49 | 1.05 | 0.72 | 0.66 | 0.22 |  | 21.03 | 0.76 | 0.93 | 0.36 |  | 21.12 | 0.94 | 0.31 |  | 19.92 | 1.20 | 19.27 | 0.27 | 0.30 |  | 19.55 | 0.40 |
| MoyTint |  | 23.73 | 0.92 | 0.33 | 0.38 | 0.63 |  | 24.55 | 0.67 | 0.93 | 0.20 |  | 24.62 | 0.83 | 0.17 |  | 23.18 | 1.05 | 25.23 | 0.25 | 0.44 |  | 25.42 | 0.34 |
| DiffTint |  | 4.61 | 1.04 | **0.03** | **0.04** | 0.18 |  | 6.89 | 0.72 | 0.95 | 0.63 |  | 6.95 | 0.93 | 0.59 |  | 6.31 | 1.19 | 12.16 | 0.74 | 0.54 |  | 12.62 | 0.70 |
| Dtmoyabs |  | 2.78 | 0.66 | 0.92 | 0.38 | 0.10 |  | 2.19 | 0.45 | 0.28 | 0.31 |  | 2.84 | 0.59 | **0.06** |  | 1.41 | 0.75 | 0.90 | 0.11 | **0.00** |  | 1.30 | 0.10 |
| Dtmaxabs |  | 5.48 | 1.29 | 0.89 | 0.63 | **0.09** |  | 4.85 | 0.88 | 0.48 | 0.17 |  | 5.66 | 1.15 | **0.05** |  | 2.76 | 1.47 | 2.34 | 0.36 | **0.01** |  | 3.32 | 0.31 |
| Maxhum |  | 96.25 | 5.06 | 0.10 | **0.06** | 0.35 |  | 86.50 | 3.41 | 0.73 | 0.50 |  | 88.08 | 4.52 | 0.68 |  | 90.45 | 5.76 | 69.45 | 1.71 | 0.57 |  | 70.44 | 1.83 |
| Minhum |  | 83.92 | 6.93 | **0.01** | **0.01** | **0.02** |  | 64.16 | 4.69 | 0.77 | 0.86 |  | 65.98 | 6.19 | 0.68 |  | 62.80 | 7.90 | 39.31 | 1.63 | 0.25 |  | 41.21 | 1.73 |
| Moyhum |  | 91.38 | 6.05 | **0.05** | **0.02** | **0.08** |  | 76.97 | 4.06 | 0.67 | 0.83 |  | 79.27 | 5.40 | 0.90 |  | 78.45 | 6.88 | 54.46 | 1.34 | 0.32 |  | 55.83 | 1.50 |
| Diffhum |  | 11.72 | 3.15 | **0.00** | **0.00** | **0.00** |  | 21.55 | 2.25 | 0.93 | 0.20 |  | 21.30 | 2.81 | 0.16 |  | 26.29 | 3.61 | 30.08 | 2.15 | 0.72 |  | 29.30 | 2.03 |

Table S4: P-values of covariates of the different models considered in a backward model selection procedure for the effect on 10 microclimate parameters of nest type (Type), presence of birds in the nest (Occupation), number of days between the first egg-laying date of breeding European rollers and the day of measurement (Developmental stage) and the Julian calendar date (Date), measured in natural cavities and nest-boxes in Vallée des Baux (France). Models are Linear Mixed Models with Julian calendar date and Nest identity as random factors. The model selection procedure started from the full model and the factor with highest p-value was removed until all remaining factors have a p-value<0.05. MaxTint: daily maximal temperature in the nest. MinTint: daily minimal temperature in the nest. MoyTint: daily mean temperature in the nest. DiffTint: difference between daily maximal and minimal temperature in the nest. Dtmaxabs: absolute value of the daily maximum delta between interior and exterior temperature. Dtmoyabs: mean over a day of the absolute delta between interior and exterior temperature. Maxhum: daily maximum humidity level in the nest. Minhum: daily minimum humidity level in the nest. Moyhum: daily mean humidity level in the nest. DiffHum: difference between daily maximal and minimal humidity level in the nest.

| **Covariates** |  | **Date** |  | **Developmental stage** |  | **Occupation*Type** |  | **Occupation** |  | **Type** |
| --- | --- | --- | --- | --- | --- | --- | --- | --- | --- | --- |
| MaxTint |  | 0.888 |  | 0.203 |  | <0.01 |  | <0.01 |  | <0.01 |
|  |  |  |  | 0.199 |  | <0.01 |  | <0.01 |  | <0.01 |
|  |  |  |  |  |  | <0.01 |  | <0.01 |  | <0.01 |
|  |  |  |  |  |  |  |  |  |  |  |
| MinTint |  | 0.214 |  | 0.061 |  | <0.01 |  | <0.01 |  | <0.01 |
|  |  |  |  | 0.102 |  | <0.01 |  | <0.01 |  | <0.01 |
|  |  |  |  |  |  | <0.01 |  | <0.01 |  | <0.01 |
|  |  |  |  |  |  |  |  |  |  |  |
| MoyTint |  | 0.625 |  | 0.025 |  | <0.01 |  | <0.01 |  | 0.013 |
|  |  |  |  | 0.029 |  | <0.01 |  | <0.01 |  | 0.014 |
|  |  |  |  |  |  |  |  |  |  |  |
| DiffTint |  | 0.152 |  | 0.969 |  | 0.631 |  | 0.298 |  | <0.01 |
|  |  | 0.135 |  |  |  | 0.631 |  | 0.297 |  | <0.01 |
|  |  | 0.215 |  |  |  |  |  |  |  | <0.01 |
|  |  |  |  |  |  |  |  |  |  | <0.01 |
|  |  |  |  |  |  |  |  |  |  |  |
| Dtmoyabs |  | 0.217 |  | 0.824 |  | <0.01 |  | <0.01 |  | <0.01 |
|  |  | 0.213 |  |  |  | <0.01 |  | <0.01 |  | <0.01 |
|  |  |  |  |  |  | <0.01 |  | <0.01 |  | <0.01 |
|  |  |  |  |  |  |  |  |  |  |  |
| Dtmaxabs |  | 0.201 |  | 0.883 |  | <0.01 |  | <0.01 |  | <0.01 |
|  |  | 0.144 |  |  |  | <0.01 |  | <0.01 |  | <0.01 |
|  |  |  |  |  |  | <0.01 |  | <0.01 |  | <0.01 |
|  |  |  |  |  |  |  |  |  |  |  |
| MaxHum |  | 0.967 |  | <0.01 |  | <0.01 |  | <0.01 |  | <0.01 |
|  |  |  |  | <0.01 |  | <0.01 |  | <0.01 |  | <0.01 |
|  |  |  |  |  |  |  |  |  |  |  |
| MinHum |  | 0.941 |  | 0.091 |  | 0.034 |  | 0.659 |  | <0.01 |
|  |  |  |  | 0.072 |  | 0.034 |  | 0.663 |  | <0.01 |
|  |  |  |  | 0.083 |  | 0.047 |  |  |  | <0.01 |
|  |  |  |  |  |  | 0.059 |  |  |  | <0.01 |
|  |  |  |  |  |  |  |  |  |  | <0.01 |
|  |  |  |  |  |  |  |  |  |  |  |
| MoyHum |  | 0.871 |  | <0.01 |  | <0.01 |  | <0.01 |  | <0.01 |
|  |  |  |  | <0.01 |  | <0.01 |  | <0.01 |  | <0.01 |
|  |  |  |  |  |  |  |  |  |  |  |
| DiffHum |  | 0.446 |  | 0.86 |  | 0.011 |  | 0.039 |  | <0.01 |
|  |  | 0.392 |  |  |  | 0.011 |  | 0.041 |  | <0.01 |
|  |  |  |  |  |  | <0.01 |  | 0.017 |  | <0.01 |

Table S5: Effect of cavity type, nest occupation and development stage of the clutch (only for nests of European rollers) during monitoring on different daily microclimate variables, in natural cavities and artificial nest-boxes available for European roller reproduction in the Vallée des Baux (France). Estimates and probabilities are from the best models of the model selection (see Table S3). MaxTint: daily maximal temperature in the nest. MinTint: daily minimal temperature in the nest. MoyTint: daily mean temperature in the nest. DiffTint: difference between daily maximal and minimal temperature in the nest. Dtmaxabs: absolute value of the daily maximum delta between interior and exterior temperature. Dtmoyabs: mean over a day of the absolute delta between interior and exterior temperature. Maxhum: daily maximum humidity level in the nest. Minhum: daily minimum humidity level in the nest. Moyhum: daily mean humidity level in the nest. DiffHum: difference between daily maximal and minimal humidity level in the nest. “CI”: 95% confidence interval of the estimate. “t”: t-value for the corresponding estimate. “*P”*: p-value. “n”: sample size. *P*-values lower than 0.10 are in bold.

| Response variable |  | Empty cavities  (n = 22) | |  | Empty nest-boxes  (n = 19) | |  | Occupied cavities  (n = 21) | |  | Occupied nest-boxes (n = 18) | |  | Developmental stage | | Paired t-tests | | | | | | | | | | |
| --- | --- | --- | --- | --- | --- | --- | --- | --- | --- | --- | --- | --- | --- | --- | --- | --- | --- | --- | --- | --- | --- | --- | --- | --- | --- | --- |
|  |  |  |  |  |  |  |  |  |  |  |  |  |  |  |  | Empty nest-boxes and cavities | |  | Occupied nest-boxes and cavities | |  | Occupied and empty nest-boxes | |  | Occupied and empty cavities | |
|  |  | Estimate (CI) | |  | Estimate (CI) | |  | Estimate (CI) | |  | Estimate (CI) | |  | Estimate (CI) | | t | *P* |  | t | *P* |  | t | *P* |  | t | *P* |
| MaxTint |  | 27.47 | [26.53;28.43] |  | 31.73 | [30.74;32.76] |  | 31.29 | [30.31;32.24] |  | 32.33 | [31.31;33.39] |  | - | - | 7.31 | **<0.01** |  | -1.89 | **0.06** |  | -1.81 | **0.07** |  | 9.24 | **<0.01** |
| MinTint |  | 20.86 | [20.01;21.70] |  | 19.35 | [18.48;20.23] |  | 25.16 | [24.28;26.01] |  | 20.12 | [19.22;21.01] |  | - | - | -3.12 | **<0.01** |  | 10.48 | **<0.01** |  | -2.78 | **<0.01** |  | 12.67 | **<0.01** |
| MoyTint |  | 24.45 | [23.63;25.26] |  | 25.53 | [24.69;26.34] |  | 28.58 | [27.75;29.38] |  | 25.98 | [25.17;26.78] |  | -0.02 | [-0.03;0.00] | 2.52 | **0.01** |  | 6.00 | **<0.01** |  | -2.26 | **0.02** |  | 15.78 | **<0.01** |
| DiffTint |  | 6.34 | [5.50;7.16] |  | 12.31 | [11.42;13.20] |  | 6.34 | [5.50;7.16] |  | 12.31 | [11.42;13.20] |  | - | - | 11.46 | **<0.01** |  | 11.46 | **<0.01** |  | NA | NA |  | NA | NA |
| Dtmoyabs |  | 2.48 | [2.06;2.90] |  | 1.12 | [0.68;1.54] |  | 4.59 | [4.17;5.02] |  | 1.63 | [1.19;2.07] |  | - | - | -4.59 | **<0.01** |  | 9.86 | **<0.01** |  | -3.26 | **<0.01** |  | 10.57 | **<0.01** |
| Dtmaxabs |  | 5.11 | [4.29;5.92] |  | 2.85 | [1.20;3.70] |  | 9.18 | [8.33;10.00] |  | 3.74 | [2.88;4.60] |  | - | - | -3.65 | **<0.01** |  | 9.07 | **<0.01** |  | -2.82 | **<0.01** |  | 10.31 | **<0.01** |
| Maxhum |  | 89.40 | [85.50;93.26] |  | 66.84 | [63.10;70.63] |  | 82.50 | [78.62;86.34] |  | 73.54 | [69.77;77.43] |  | 0.15 | [0.07;0.23] | -9.63 | **<0.01** |  | 3.80 | **<0.01** |  | -5.44 | **<0.01** |  | -4.37 | **<0.01** |
| Minhum |  | 71.37 | [67.04;75.71] |  | 41.84 | [37.21;46.48] |  | 71.37 | [67.04;75.71] |  | 41.84 | [37.21;46.48] |  | - | - | -9.77 | **<0.01** |  | -9.77 | **<0.01** |  | NA | NA |  | NA | NA |
| Moyhum |  | 81.25 | [77.18;85.30] |  | 52.67 | [48.59;56.79] |  | 77.16 | [73.17;81.32] |  | 58.84 | [54.81;62.98] |  | 0.13 | [0.05;0.21] | -11.07 | **<0.01** |  | 7.08 | **<0.01** |  | -5.49 | **<0.01** |  | -2.76 | **<0.01** |
| DiffHum |  | 20.22 | [17.16;23.21] |  | 29.71 | [26.68;32.87] |  | 16.34 | [13.28;19.48] |  | 31.58 | [28.40;34.83] |  | - | - | 4.95 | **<0.01** |  | -7.58 | **<0.01** |  | -1.30 | 0.20 |  | -2.09 | **0.04** |

Table S6: Estimates of the interaction effect of mean microclimate variables over 15 consecutive days with nest type (natural cavities or artificial nest-boxes) on: breeding parameters (number of fledglings, clutch size, probability that a nest was successful and probability that an egg produced a fledging) and probability of a nest being used by European rollers in the Vallée des Baux (France). MAXTint: mean of the daily maximal temperature in the nest. MINTint: mean of the daily minimal temperature in the nest. MOYTint: mean of the daily mean temperature in the nest. DIFFTint: mean of the difference between daily maximal and minimal temperature in the nest. DTMAXabs: mean of the absolute value of the daily maximum delta between interior and exterior temperature. DTMOYabs: mean of the mean over a day of the absolute delta between interior and exterior temperature. MAXHum: mean of the daily maximum humidity level in the nest. MINHum: mean of the daily minimum humidity level in the nest. MOYHum: mean of the daily mean humidity level in the nest. DIFFHum: mean of the difference between daily maximal and minimal humidity level in the nest. “n”: number of nest-boxes. “N”: number of natural cavities. “SE”: standard error of the estimate. “Z”: z-value for the corresponding estimate. “*P”*: p-value. “n”: sample size. *P*-values lower than 0.10 are in bold. a1, a2, b1 and b2 are the coefficients of the model equation y = a_1_ + a_2_ * W + b_1_ * X + b_2_ * X * W, for any response variable “y” and any microclimate variable “X” with W = 0 for natural cavities and W =1 for artificial nest-boxes.

| Explanatory variables | Model coefficients | Response variables | | | | | | | | | | | | | | | | | | | | | | | |
| --- | --- | --- | --- | --- | --- | --- | --- | --- | --- | --- | --- | --- | --- | --- | --- | --- | --- | --- | --- | --- | --- | --- | --- | --- | --- |
|  |  | Number of fledglings (n=15; N=16) | | | |  | Clutch size (n=15; N=16) | | | |  | Probability that a nest was successful (n=17; N=20) | | | |  | Probability that an egg produced a fledgling  (n=15; N=16) | | | |  | Probability of use by rollers (n=33; N=41) | | | |
|  |  | Estimate | SE | Z | *p* |  | Estimate | SE | Z | *p* |  | Estimate | SE | Z | *p* |  | Estimate | SE | Z | *p* |  | Estimate | SE | Z | *p* |
| MAXTint | a1 | 1.51 | 0.15 | 10.39 | <0.01 |  | 1.64 | 0.14 | 11.99 | <0.01 |  | 1.17 | 0.59 | 1.98 | 0.05 |  | 2.02 | 0.46 | 4.40 | <0.01 |  | -7.38 | 4.56 | -1.62 | 0.11 |
|  | b1 | 0.17 | 0.18 | 0.96 | 0.34 |  | 0.11 | 0.17 | 0.66 | 0.51 |  | -0.64 | 0.65 | -0.98 | 0.33 |  | 0.51 | 0.56 | 0.92 | 0.36 |  | 0.26 | 0.16 | 1.65 | **0.10** |
|  | a2 | -0.13 | 0.21 | -0.63 | 0.53 |  | -0.05 | 0.20 | -0.27 | 0.78 |  | 1.66 | 1.45 | 1.14 | 0.26 |  | -0.58 | 0.58 | -1.00 | 0.32 |  | -8.10 | 8.73 | -0.93 | 0.35 |
|  | b2 | -0.24 | 0.24 | -1.00 | 0.32 |  | -0.17 | 0.22 | -0.77 | 0.44 |  | -0.29 | 1.19 | -0.25 | 0.81 |  | -0.57 | 0.66 | -0.85 | 0.39 |  | 0.22 | 0.28 | 0.80 | 0.42 |
|  |  |  |  |  |  |  |  |  |  |  |  |  |  |  |  |  |  |  |  |  |  |  |  |  |  |
| MINTint | a1 | 1.39 | 0.18 | 7.76 | <0.01 |  | 1.59 | 0.16 | 9.85 | <0.01 |  | 0.94 | 0.64 | 1.47 | 0.14 |  | 1.54 | 0.41 | 3.76 | <0.01 |  | 0.05 | 0.38 | 0.13 | 0.90 |
|  | b1 | 0.05 | 0.14 | 0.36 | 0.72 |  | 0.00 | 0.13 | 0.00 | 0.99 |  | 1.49 | 0.86 | 1.73 | **0.08** |  | 0.32 | 0.35 | 0.92 | 0.36 |  | 0.17 | 0.36 | 0.47 | 0.64 |
|  | a2 | -0.06 | 0.29 | -0.22 | 0.83 |  | -0.06 | 0.26 | -0.22 | 0.83 |  | 1.61 | 1.53 | 1.06 | 0.29 |  | -0.07 | 0.66 | -0.10 | 0.92 |  | 0.72 | 0.70 | 1.03 | 0.30 |
|  | b2 | -0.06 | 0.31 | -0.18 | 0.86 |  | -0.03 | 0.28 | -0.10 | 0.92 |  | -0.78 | 1.63 | -0.48 | 0.63 |  | -0.22 | 0.72 | -0.30 | 0.76 |  | 0.81 | 0.74 | 1.09 | 0.27 |
|  |  |  |  |  |  |  |  |  |  |  |  |  |  |  |  |  |  |  |  |  |  |  |  |  |  |
| MOYTint | a1 | 1.41 | 0.14 | 10.08 | <0.01 |  | 1.58 | 0.13 | 12.43 | <0.01 |  | 1.38 | 0.57 | 2.42 | 0.02 |  | 1.70 | 0.35 | 4.90 | <0.01 |  | -5.68 | 4.72 | -1.21 | 0.23 |
|  | b1 | 0.07 | 0.10 | 0.69 | 0.49 |  | 0.03 | 0.09 | 0.33 | 0.74 |  | 0.35 | 0.53 | 0.66 | 0.51 |  | 0.28 | 0.28 | 0.98 | 0.33 |  | 0.22 | 0.18 | 1.24 | 0.22 |
|  | a2 | -0.08 | 0.20 | -0.39 | 0.70 |  | -0.03 | 0.18 | -0.17 | 0.86 |  | 0.62 | 0.95 | 0.65 | 0.52 |  | -0.30 | 0.46 | -0.64 | 0.52 |  | -20.55 | 11.91 | -1.73 | **0.08** |
|  | b2 | -0.10 | 0.26 | -0.37 | 0.71 |  | -0.05 | 0.23 | -0.22 | 0.83 |  | -1.00 | 1.53 | -0.66 | 0.51 |  | -0.31 | 0.58 | -0.53 | 0.59 |  | 0.79 | 0.46 | 1.73 | **0.08** |
|  |  |  |  |  |  |  |  |  |  |  |  |  |  |  |  |  |  |  |  |  |  |  |  |  |  |
| DIFFTint | a1 | 1.57 | 0.26 | 60.3 | <0.01 |  | 1.75 | 0.24 | 7.27 | <0.01 |  | 0.02 | 0.90 | 0.02 | 0.98 |  | 1.64 | 0.69 | 2.39 | 0.02 |  | 0.69 | 0.54 | 1.28 | 0.20 |
|  | b1 | 0.17 | 0.29 | 0.58 | 0.56 |  | 0.19 | 0.27 | 0.71 | 0.48 |  | -3.01 | 1.58 | -1.91 | **0.06** |  | -0.17 | 0.80 | -0.22 | 0.83 |  | 0.74 | 0.58 | 1.28 | 0.20 |
|  | a2 | -0.18 | 0.33 | -0.55 | 0.58 |  | -0.15 | 0.30 | -0.50 | 0.62 |  | 3.10 | 1.81 | 1.71 | **0.09** |  | -0.17 | 0.82 | -0.21 | 0.83 |  | -1.19 | 0.84 | -1.42 | 0.16 |
|  | b2 | -0.24 | 0.35 | -0.68 | 0.50 |  | -0.25 | 0.32 | -0.76 | 0.45 |  | 1.96 | 1.92 | 1.02 | 0.31 |  | 0.09 | 0.91 | 0.10 | 0.92 |  | -0.10 | 0.85 | -0.12 | 0.91 |
|  |  |  |  |  |  |  |  |  |  |  |  |  |  |  |  |  |  |  |  |  |  |  |  |  |  |
| DTMAXabs | a1 | 1.43 | 0.15 | 9.43 | <0.01 |  | 1.55 | 0.14 | 10.77 | <0.01 |  | 1.20 | 0.59 | 2.04 | 0.04 |  | 2.10 | 0.49 | 4.32 | <0.01 |  | -0.60 | 1.05 | -0.58 | 0.56 |
|  | b1 | 0.02 | 0.14 | 0.16 | 0.87 |  | 0.09 | 0.13 | 0.65 | 0.52 |  | 0.55 | 0.65 | 0.84 | 0.40 |  | -0.45 | 0.40 | -1.12 | 0.26 |  | 0.11 | 0.14 | 0.75 | 0.45 |
|  | a2 | -0.05 | 0.22 | -0.23 | 0.82 |  | 0.05 | 0.20 | 0.23 | 0.82 |  | 1.28 | 1.22 | 1.05 | 0.29 |  | -0.69 | 0.60 | -1.14 | 0.25 |  | -3.21 | 1.92 | -1.68 | **0.09** |
|  | b2 | 0.05 | 0.21 | 0.22 | 0.83 |  | -0.02 | 0.19 | -0.11 | 0.91 |  | -1.51 | 1.31 | -1.16 | 0.25 |  | 0.47 | 0.52 | 0.90 | 0.37 |  | 1.09 | 0.55 | 1.98 | **0.05** |
|  |  |  |  |  |  |  |  |  |  |  |  |  |  |  |  |  |  |  |  |  |  |  |  |  |  |
| DTMOYabs | a1 | 1.41 | 0.18 | 7.89 | <0.01 |  | 1.53 | 0.17 | 9.04 | <0.01 |  | 0.88 | 0.64 | 1.39 | 0.17 |  | 2.10 | 0.54 | 3.89 | <0.01 |  | -0.15 | 0.88 | -0.17 | 0.87 |
|  | b1 | 0.03 | 0.14 | 0.21 | 0.84 |  | 0.07 | 0.13 | 0.58 | 0.56 |  | 1.58 | 0.92 | 1.71 | **0.09** |  | -0.32 | 0.37 | -0.86 | 0.39 |  | 0.08 | 0.24 | 0.36 | 0.72 |
|  | a2 | 0.27 | 0.40 | 0.67 | 0.50 |  | 0.25 | 0.37 | 0.68 | 0.50 |  | 0.49 | 2.67 | 0.19 | 0.85 |  | -0.07 | 1.02 | -0.07 | 0.94 |  | -5.78 | 2.26 | -2.55 | **0.01** |
|  | b2 | 0.43 | 0.47 | 0.91 | 0.36 |  | 0.22 | 0.43 | 0.52 | 0.60 |  | -3.49 | 3.87 | -0.90 | 0.37 |  | 1.13 | 1.08 | 1.04 | 0.30 |  | 4.49 | 1.62 | 2.76 | **<0.01** |
|  |  |  |  |  |  |  |  |  |  |  |  |  |  |  |  |  |  |  |  |  |  |  |  |  |  |
| MAXHum | a1 | 1.44 | 0.21 | 6.81 | <0.01 |  | 1.64 | 0.19 | 8.61 | <0.01 |  | 0.84 | 0.78 | 1.08 | 0.28 |  | 1.48 | 0.51 | 2.91 | <0.01 |  | 0.39 | 0.39 | 0.99 | 0.32 |
|  | b1 | -0.00 | 0.19 | -0.01 | 0.99 |  | -0.05 | 0.17 | -0.30 | 0.77 |  | 2.19 | 0.98 | 2.24 | **0.03** |  | 0.36 | 0.49 | 0.74 | 0.46 |  | -0.39 | 0.36 | -1.09 | 0.28 |
|  | a2 | -0.15 | 0.32 | -0.47 | 0.64 |  | -0.10 | 0.29 | -0.35 | 0.72 |  | 1.74 | 1.84 | 0.95 | 0.34 |  | -0.21 | 0.72 | -0.30 | 0.77 |  | -0.09 | 0.81 | -0.11 | 0.91 |
|  | b2 | -0.06 | 0.34 | -0.19 | 0.85 |  | 0.03 | 0.31 | 0.08 | 0.93 |  | -1.41 | 2.11 | -0.67 | 0.50 |  | -0.57 | 0.81 | -0.70 | 0.48 |  | 0.71 | 0.89 | 0.80 | 0.42 |
|  |  |  |  |  |  |  |  |  |  |  |  |  |  |  |  |  |  |  |  |  |  |  |  |  |  |
| MINHum | a1 | 1.42 | 0.22 | 6.34 | <0.01 |  | 1.60 | 0.20 | 7.86 | <0.01 |  | 0.68 | 0.79 | 0.86 | 0.39 |  | 1.58 | 0.56 | 2.81 | <0.01 |  | 0.25 | 0.40 | 0.62 | 0.53 |
|  | b1 | 0.02 | 0.19 | 0.12 | 0.91 |  | -0.01 | 0.18 | -0.05 | 0.96 |  | 2.43 | 1.07 | 2.27 | **0.02** |  | 0.22 | 0.52 | 0.43 | 0.66 |  | -0.16 | 0.37 | -0.42 | 0.68 |
|  | a2 | 0.21 | 0.43 | 0.49 | 0.62 |  | 0.12 | 0.40 | 0.30 | 0.76 |  | 3.07 | 2.55 | 1.21 | 0.23 |  | 0.50 | 1.07 | 0.47 | 0.64 |  | 1.61 | 1.23 | 1.31 | 0.19 |
|  | b2 | 0.38 | 0.52 | 0.73 | 0.47 |  | 0.23 | 0.47 | 0.50 | 0.62 |  | -0.38 | 2.72 | -0.14 | 0.89 |  | 0.67 | 1.22 | 0.55 | 0.58 |  | 2.31 | 1.36 | 1.70 | **0.09** |
|  |  |  |  |  |  |  |  |  |  |  |  |  |  |  |  |  |  |  |  |  |  |  |  |  |  |
| DIFFHum | a1 | 1.40 | 0.21 | 6.73 | <0.01 |  | 1.54 | 0.19 | 8.03 | <0.01 |  | 0.78 | 0.67 | 1.17 | 0.24 |  | 1.51 | 0.56 | 2.72 | <0.01 |  | -0.01 | 0.38 | -0.04 | 0.97 |
|  | b1 | -0.05 | 0.20 | -0.27 | 0.79 |  | -0.06 | 0.18 | -0.34 | 0.73 |  | -1.59 | 0.86 | -1.84 | **0.07** |  | 0.30 | 0.52 | 0.58 | 0.56 |  | -0.28 | 0.38 | -0.74 | 0.46 |
|  | a2 | 0.16 | 0.30 | 0.54 | 0.59 |  | 0.13 | 0.27 | 0.46 | 0.65 |  | 1.77 | 1.51 | 1.17 | 0.24 |  | 0.00 | 0.94 | 0.00 | 1.00 |  | 0.59 | 0.66 | 0.90 | 0.37 |
|  | b2 | -0.30 | 0.33 | -0.84 | 0.40 |  | -0.11 | 0.30 | -0.35 | 0.72 |  | 0.94 | 1.49 | 0.63 | 0.53 |  | -0.17 | 1.08 | -0.15 | 0.88 |  | -0.44 | 0.68 | -0.64 | 0.52 |
|  |  |  |  |  |  |  |  |  |  |  |  |  |  |  |  |  |  |  |  |  |  |  |  |  |  |
| MOYHum | a1 | 1.43 | 0.22 | 6.37 | <0.01 |  | 1.63 | 0.20 | 7.98 | <0.01 |  | 0.75 | 0.82 | 0.91 | 0.36 |  | 1.83 | 0.56 | 3.29 | <0.01 |  | 0.36 | 0.41 | 0.88 | 0.38 |
|  | b1 | 0.00 | 0.19 | 0.02 | 0.98 |  | -0.04 | 0.18 | -0.21 | 0.84 |  | 2.43 | 1.04 | 2.34 | **0.02** |  | 0.06 | 0.53 | 0.12 | 0.90 |  | -0.31 | 0.38 | -0.83 | 0.40 |
|  | a2 | -0.07 | 0.41 | -0.18 | 0.86 |  | -0.07 | 0.37 | -0.19 | 0.85 |  | 2.88 | 2.65 | 1.09 | 0.28 |  | 0.24 | 0.80 | 0.30 | 0.76 |  | 1.17 | 1.19 | 0.99 | 0.32 |
|  | b2 | 0.03 | 0.47 | 0.07 | 0.94 |  | 0.04 | 0.42 | 0.11 | 0.92 |  | -0.47 | 2.89 | -0.16 | 0.87 |  | -0.98 | 0.82 | -1.20 | 0.23 |  | 2.10 | 1.32 | 1.58 | 0.11 |
